# Diversification and evolution of the *SDG* gene family in *Brassica rapa* after the whole genome triplication

**DOI:** 10.1038/srep16851

**Published:** 2015-11-24

**Authors:** Heng Dong, Dandan Liu, Tianyu Han, Yuxue Zhao, Ji Sun, Sue Lin, Jiashu Cao, Zhong-Hua Chen, Li Huang

**Affiliations:** 1 Laboratory of Cell & Molecular Biology, Institute of Vegetable Science, Zhejiang University, Hangzhou, 310058, China; 2 Key Laboratory of Horticultural Plant Growth, Development and Quality Improvement, Ministry of Agriculture, Hangzhou, 310058, China; 3 Zhejiang Provincial Key Laboratory of Horticultural Plant Integrative Biology Hangzhou, 310058, China; 4 Wenzhou Vocational College of Science and Technology, Wenzhou, 325006, China; 5 Department of Agronomy, Zhejiang Key Laboratory of Crop Germplasm, Zhejiang University, Hangzhou, 310058, China; 6 School of Science and Health, Western Sydney University, Penrith, NSW 2751, Australia

## Abstract

Histone lysine methylation, controlled by the SET Domain Group (SDG) gene family, is part of the histone code that regulates chromatin function and epigenetic control of gene expression. Analyzing the *SDG* gene family in *Brassica rapa* for their gene structure, domain architecture, subcellular localization, rate of molecular evolution and gene expression pattern revealed common occurrences of subfunctionalization and neofunctionalization in *BrSDGs*. In comparison with *Arabidopsis thaliana*, the *BrSDG* gene family was found to be more divergent than *AtSDGs*, which might partly explain the rich variety of morphotypes in *B. rapa*. In addition, a new evolutionary pattern of the four main groups of *SDGs* was presented, in which the Trx group and the SUVR subgroup evolved faster than the E(z), Ash groups and the SUVH subgroup. These differences in evolutionary rate among the four main groups of *SDG*s are perhaps due to the complexity and variability of the regions that bind with biomacromolecules, which guide *SDGs* to their target loci.

Histone lysine methylation plays critical roles in the epigenetic regulation of gene expression^1^. It participates in plant growth and development, also exhibits dynamic changes to important environmental factors, such as hormones, water-stress and light^2^,^3^,^4^. In plants, histone lysine methylation occurs at several residues, including four (K4, K9, K27, K36) on H3 and one (K20) on H4. All these lysines can be mono-, di- or tri-methylated, increasing the complexity of epigenetic modification.

Histone lysine methylation depends on histone lysine methyltransferases (HKMTases) and in plants the SET Domain Group (SDG) protein family, named after three *Drosophila melanogaster* proteins (Suppressor variegation 3–9, Enhancer of Zeste and Trithorax), is believed to be the only HKMTase family. The *SDG* gene family is classified into seven groups in *Arabidopsis thaliana*: Group I, *Enhancer of zeste* homologs [E(z)]; Group II, *Ash1* homologs and related (Ash); Group III, *trithorax* (*trx*) homologs and related (Trx); Group IV, *Arabidopsis trx related 5* (*ATXR5*) and *ATXR6* homologs (ATXR5/6); Group V, *Suppressor of variegation* [*Su(var)*] homologs and related (Suv); Group VI, SET- and myeloid-Nervy-DEAF-1 (MYND)-domain containing HKMTases (SMYD); Group VII, RBCMT and other SET-related proteins (SETD). The E(z), Ash, Trx and Suv groups are treated as the four main groups^5^,^6^. In general, the E(z), ATXR5/6 and Suv proteins play a role in repressing gene/transposon expression through accumulating H3K27 or H3K9 methylation modifications, while the Ash and Trx proteins methylate H3K36 and H3K4 thereby activating gene expression^7^.

*AtSDGs* are the best functional characterized *SDG* gene family and a growing body of work has illustrated that SDG proteins in different groups maybe involved in similar processes. For example, in *A. thaliana*, one E(z) protein, CURLYLEAF (CLF), four Trx proteins, Arabidopsis trx1 (ATX1), ATX2, ATXR3 and ATXR7, and two Ash proteins, ASH1-HOMOLOG1 (ASHH1) and ASHH2 all act synergistically to regulate flowering time through controlling the expression of *FLOWERING LOCUS C* (*FLC*)^8^,^9^,^10^,^11^,^12^,^13^,^14^. ASH1-RELATED3 (ASHR3), ASHH2 in the Ash group, ATXR3 in the Trx group and ATXR6 in the ATXR5/6 group are required for sporophyte development^15^,^16^,^17^,^18^.

Studies have been performed on the *SDG* gene family in other species such as *Oryza sativa*, *Zea mays*, *Vitis vinifera* and *Populus trichocarpa*^6^,^7^,^19^, but little is known about *SDGs* in vegetable crops. *Brassica rapa* is an important economic vegetable crop and shares a common ancestor with *A. thaliana*. A whole genome triplication (WGT) event, which occurred between 13 and 17 million years ago, distinguished its genome from that of *A. thaliana*^20^. This time span is long enough for the genome to be fractionated but short enough for most of the genes to be clearly identified in *A. thaliana*, making *B. rapa* ideal for studying the expansion of gene families^21^,^22^.

In order to obtain more detailed information about the *SDG* gene family in vegetable crops, identification of the *SDGs* in the genome of *B. rapa* was carried out then the comparative analysis of them with *AtSDGs* were performed at the gene structure, domain architecture, subcellular localization, rate of molecular evolution and gene expression pattern. Sixty-seven *BrSDGs* were annotated and proved to be highly divergent. In addition, a new group evolutionary pattern among the four main groups was presented and two hypotheses were put forward to account for this. This study will shed some light for a better understanding of the evolution and the function of the *SDG* gene family in vegetable crops.

## Results

### Identification of *BrSDGs* in the genome of *B. rapa*

**Figure 1:**
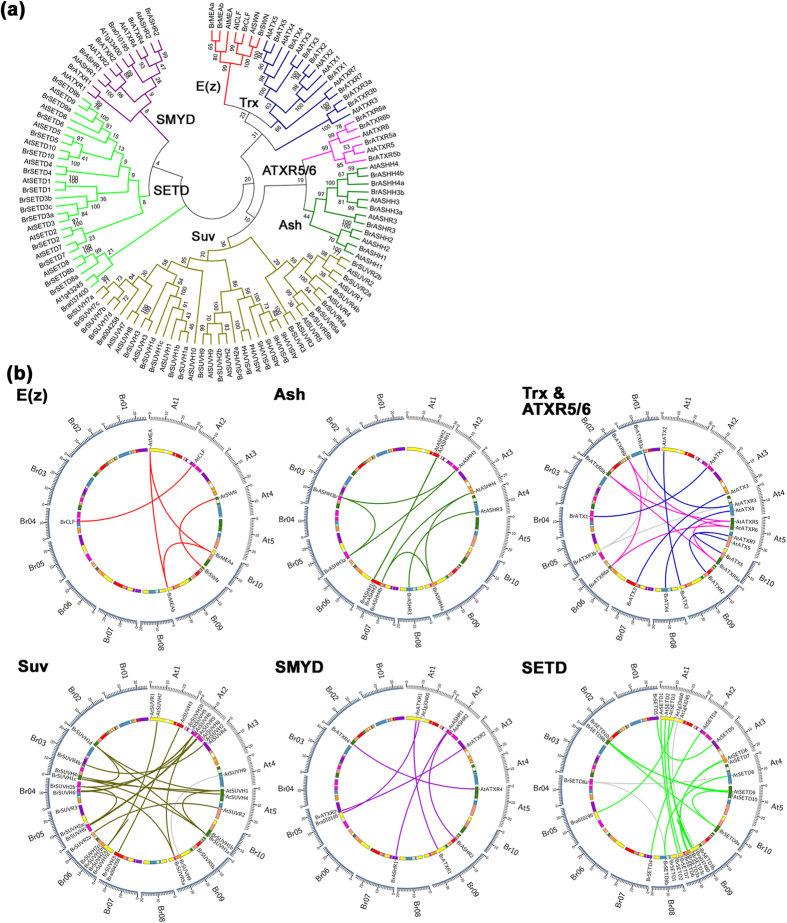
Phylogenetic and syntenic analyses for *SDGs* in *Brassica rapa* and *Arabidopsis thaliana*. (**a**) Neighbor-joining (NJ) tree of *SDGs* based on SET domains and (**b**) syntenic relationships between *BrSDGs* and *AtSDGs* according to the *Brassica* database (BRAD) are displayed. Genes in the same group are linked in the same color, and those genes with no clear syntenic counterparts are linked to genes with the greatest homology by grey lines.

A total of 67 *BrSDG*s were identified from the *B. rapa* genome and were named after their *A. thaliana* homologs (Table S1). Similar to previous studies, the phylogenetic analysis allowed the classification of these genes into seven major groups (Fig. 1a)^5^,^6^. *AtSETD8*, At1g43245 and their homologs were separated from the rest of the SETD genes. However, they were still regarded as SETD as they shared the similar SET domain architecture of typical SETD genes as did *AtATXR3* and its homologs. Four main groups, E(z), Ash, Trx and Suv, contained a total of 63% (42/67) *BrSDGs*, which was similar to that in *A. thaliana* (61%). Genes in the four main groups could be subdivided further into several clades (Table S1). Specifically, three clades in the E(z) group, four in the Ash group, four in the Trx group and seven in the Suv group^5^,^6^. Clade V-1, V-2, V-3 and V-5 in the Suv group constituted the Suv Homologs (SUVH) subgroup and the other three clades (V-4, V-6, V-7) were assigned to the Suv Related (SUVR) subgroup.

Homologs to Bra010195, Bra037400 and Bra004258 could not be found in the annotated *AtSDGs*. But the SET and RING-finger associated domain (SRA) and Pre-SET domain in Bra004258 were typical characteristics of the Suv genes and the phylogenetic analysis implied that Bra004258 might derive from *SUVH7* (Fig. 1a). In addition, two more *AtSDGs* (At1g33400 and At1g43245) were detected by syntenic analysis and proved to be the homologs of Bra010195 and Bra037400, respectively (Fig. 1b and Table S1).

Up to 94% of the *BrSDGs* were located in the same syntenic blocks as their corresponding *A. thaliana* homologs, except *BrATXR3b*, *BrSUVH9*, *BrSETD8a* and Bra037400 (Fig. 1b). Three tandem duplication clusters were identified, which turned out to be *BrSUVH1a*/*BrSUVH1b*, *BrSUVH7a*/*BrSUVH7b*/*BrSUVH7c*/*BrSUVH7d* and *BrSETD3a*/*BrSETD3b*/*BrSETD3c*, respectively (Fig. 1b; Table S1). Retention proportion analysis illustrated that, after the WGT event, only 44% of *BrSDG* loci were retained, similar to neighboring genes (40%) (Table S2) and randomly selected genes (45%), but significantly lower than that of core eukaryotic genes (52%) (*P* < 0.05).

### Gene structure analysis of *BrSDGs*

Among the *SDG* genes, *AtSUVH10*, *BrSUVH7b*, *BrSUVH7d* and *BrSUVR5a* varied significantly from their homologs in gene structure, domain architecture, and motif architecture of the SET domain (Figs S1-S5). Moreover, no expression was detected for these four genes. Data above indicate these genes are pseudogenes, so their information is not included in Table 1, Table 2 and Tables S3–S7. In addition, the SUVH and SUVR subgroups have different domain architectures and use unique mechanisms for H3K9 methylation, thus they are described and discussed separately.

A total of 535 introns were found in the *BrSDG* genes, with an average intron number of 8.4 per gene and an average intron length of 184.1 bp (Table S3). Among all the *BrSDG* introns, 57% were in Phase 0, 23% and 20% were in Phase 1 and Phase 2, respectively (see Methods for more detail). These data were similar to those for *AtSDG* genes, which contained 449 introns, with 9.4 introns in per gene and an average intron length of 138.7 bp. In addition, the data for the location of the introns were also similar, 59% in Phase 0, 19% in Phase 1 and 22% in Phase 2 (Table S3).

Interestingly, genes in Clade V-1, V-3, and V-5 are intronless in *A. thaliana*^6^, while half (7/14) of them contain introns in *B. rapa* (Fig. S1; Table S4). In addition, 61% (39/64) of *BrSDGs* demonstrated variation in gene structure when compared with their homologs in *A. thaliana*, including all Trx genes and most genes in the E(z), ATXR5/6, and SETD groups (Tables 1 and S4; Fig. S1). All the variant sites are located outside the regions of the SET domain and associated with intron gain/loss, exon gain/loss, and intron sliding (phase changing).

**Figure 2:**
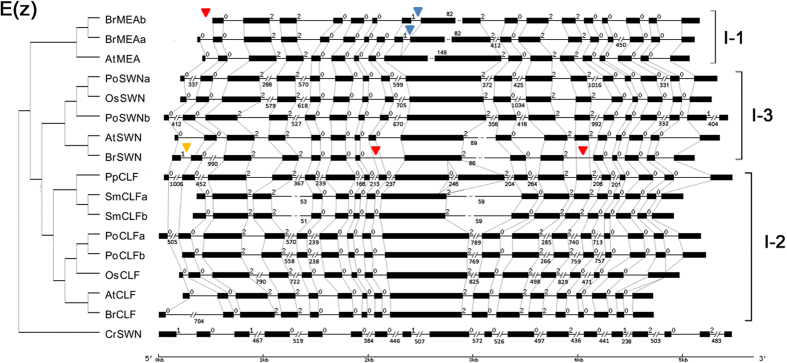
Structure of *SDG*s in the E(z) group in selected species. The species are designated as Br for *Brassica rapa*, At for *Arabidopsis thaliana*, Os for *Oryza sativa*, Pt for *Populus trichocarpa*, Sm for *Selaginella moellendorffii*, Pp for *Physcomitrella patens* and Cr for *Chlamydomonas reinhardtii*. Intron phases are shown on the introns (black lines). For the figure-sized, manually adjusted exons (black boxes) and introns, nucleotide numbers are shown above and below exons and introns, respectively. Red triangles denote changes in the exon, blue triangles denote changes in the intron, and yellow triangles denote changes in intron phase.

To determine whether these differences originated from *B. rapa* or *A. thaliana*, the gene structures of the four main groups of *SDGs* in *O. sativa* (rice), *P. trichocarpa* (poplar), *Selaginella moellendorffii* (spike moss), *Physcomitrella patens* (moss), *Chlamydomonas reinhardtii* (green alga) and *Volvox carteri* (volvox) were compared with those from *B. rapa* and *A. thaliana*. Among the 55 variant sites between the four main groups of *BrSDGs* and *AtSDGs*, 49 belonged to *BrSDGs*, with only six occurring in *AtSDGs* (Fig. 2 and S2).

### Domain architectures analysis and identification of motifs in SET domain

To better understanding the characteristics of *SDGs*, the domain architecture among the 39 deduced BrSDG proteins from the four main groups and their corresponding homologs in *A. thaliana* were investigated. Domain architecture changes were detected in 18 BrSDGs, including eight single-copy genes (Fig. S3; Table S5). Seventy-five percent of the E(z) proteins contained changes in domain architecture, while the percentages in the Trx group and the SUVR subgroup were lower (63% and 67%, respectively), followed by the SUVH subgroup (36%) and the Ash group (14%).

**Figure 3:**
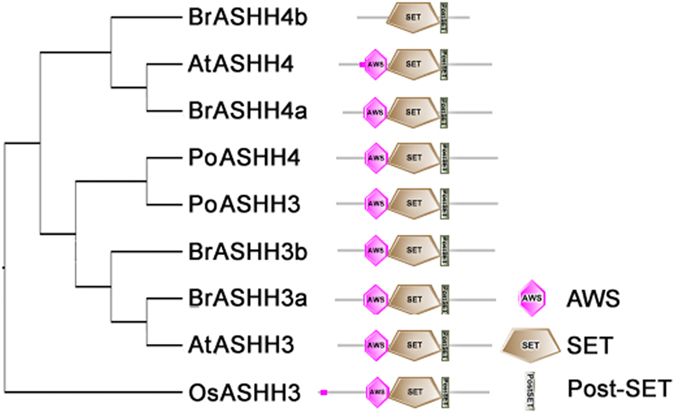
Domain architecture of *ASHH4* homologs from *Brassica rapa*, *Arabidopsis thaliana*, *Populus trichocarpa* and *Oryza sativa*. Full-length proteins were applied and searched in the Simple Modular Architecture Research Tool (SMART) and Pfam (http://pfam.xfam.org/) online databases. The name of each domain is indicated at the lower right corner. The species are designated as Br for *Brassica rapa*, At for *Arabidopsis thaliana*, Pt for *Populus trichocarpa*, and Os for *Oryza sativa*.

The analysis on the homologs of architecture-changed genes in other species demonstrated among the 30 sites with domain changes, only three changes belonged to AtSDGs, with one on AtATX4 and two on AtATXR7, while the majority was in BrSDGs. Moreover, BrMEDEA (BrMEA), BrSWINGER (BrSWN), BrASHH4b, BrATX2, BrATX3, BrATX4, BrATX5, BrSUVH1a, BrSUVH1b, BrSUVH3, BrSUVR4a, BrSUVR4b and BrSUVR5 displayed unique architecture patterns that existed only in *B. rapa* but not in other tested species (Fig. 3 and S4).

Notably, the changes in the E(z), Ash and Trx proteins represented mainly in losing domains, while SUVH and SUVR proteins gaining them. In general, the SWI3, ADA2, N-CoR and TFIII (SANT) DNA-binding domain and the cysteine-rich region (CXC) RNA binding domains were missed in two E(z) proteins respectively, and the loss of the plant homeodomain (PHD), a protein-protein interaction domain, was detected in four Trx proteins. Also, a SET structure related domain (Post-SET) was found in one Trx protein, two SUVH proteins and one SUVR protein. On the other hand, the gaining AT-hook, which binds proteins to the AT-rich DNA sequences, was identified in three SUVH proteins. Moreover, several domains were detected in SDG proteins for the first time, including the helix-hairpin-helix1 (HhH1) DNA-binding domain and the iron-sulphur binding domain (FES) finding in DNA lyase^23^,^24^ in one Trx protein and Ribosomal-14 domain for RNA binding and Stress-antifung domain for stress tolerance and antifungal activity^25^,^26^,^27^ in two SUVR proteins (Tables S5 and S6).

Using Multiple Em for Motif Elicitation (MEME), 27 conserved motifs were found in the SET domains of the BrSDGs and AtSDGs from the four main groups (Fig. S6). In contrast to the frequent variation in domain architecture, divergence in the SET domain motifs were only detected in four proteins (BrCLF, BrMEAa, BrMEAb and BrATX2) (Fig. S5).

### Subcellular localization analysis of BrSDGs and AtSDGs homologs

Nucpred and WoLF PSORT online analysis predicted that 24 BrSDGs are restricted to nucleus localization and other 25 are located in other organelles and/or the cytoplasm (Table S7). Interestingly, seven pairs of BrSDG and AtSDG homologous proteins displayed different predicted subcellular localization patterns (Table S7). Specifically, BrSUVH7a, BrSUVH7c, BrSUVR3 and BrASHH4a were predicted to be in the nucleus while their AtSDGs homologs were in the cytoplasm. In contrast, while predicted subcellular localization of BrSUVH5, BrSUVR4a and BrSUVH1a was in the cytoplasm, their corresponding AtSDGs were in the nucleus.

**Figure 4:**
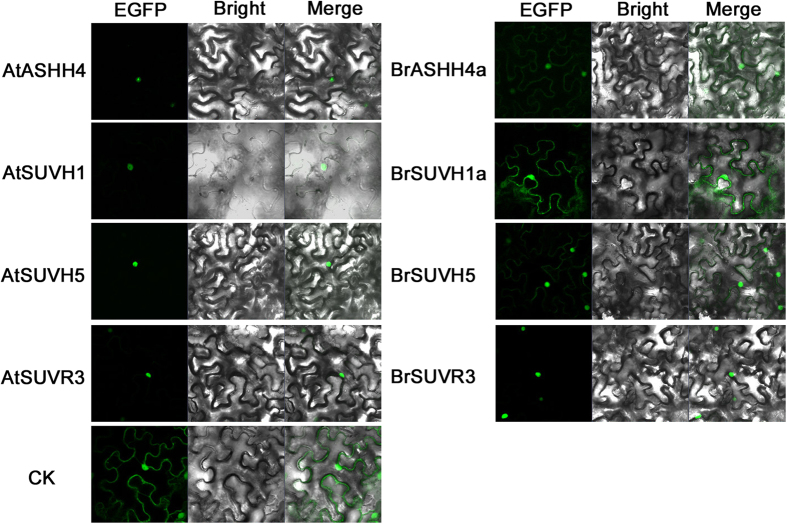
Subcellular localization of homologous *Brassica rapa* and *Arabidopsis thaliana* SDGs in tobacco leaf epidermal cells were transiently expressed using *pFGC-EGFP-SDG* fusion constructs. AtASHH4, AtSUVH1, AtSUVH5, AtSUVR3 and BrSUVR3 are concentrated in the nucleus, whereas BrASHH4a, BrSUVH1a and BrSUVH5 exhibited both cytoplasmic and nuclear localization. *pFGC-EGFP* vector was used as a control (CK).

To confirm the predicted subcellular localization, *EGFP-SDG* expression vectors were constructed and transiently expressed in tobacco leaf epidermal cells. As the expression of *BrSUVH7a* and *BrSUVH7c* were not detected in our experiments, and a full-length clone of *BrSUVR4a* could not be obtained, only four pairs of SDGs were compared. BrSUVH5 was detected in both the nucleus and cytoplasm, whereas AtSUVH5 was located exclusively in the nucleus. The BrSUVH1a protein and AtSUVH1 showed a similar subcellular localization pattern to BrSUVH5 and AtSUVH5, respectively. However, the protein pair BrASHH4a and AtASHH4 exhibited an opposite trend to the Nucpred and WoLF PSORT prediction with BrASHH4a locating in both the nucleus and cytoplasm and AtASHH4 restricting in the nucleus. Moreover, both BrSUVR3 and AtSUVR3 were located in nucleus, which was also different from the prediction (Fig. 4). The same subcellular localization results were observed in the transient expression in onion epidermal cells (data not shown).

### Analysis of molecular evolutionary rate on *BrSDGs*

**Figure 5:**
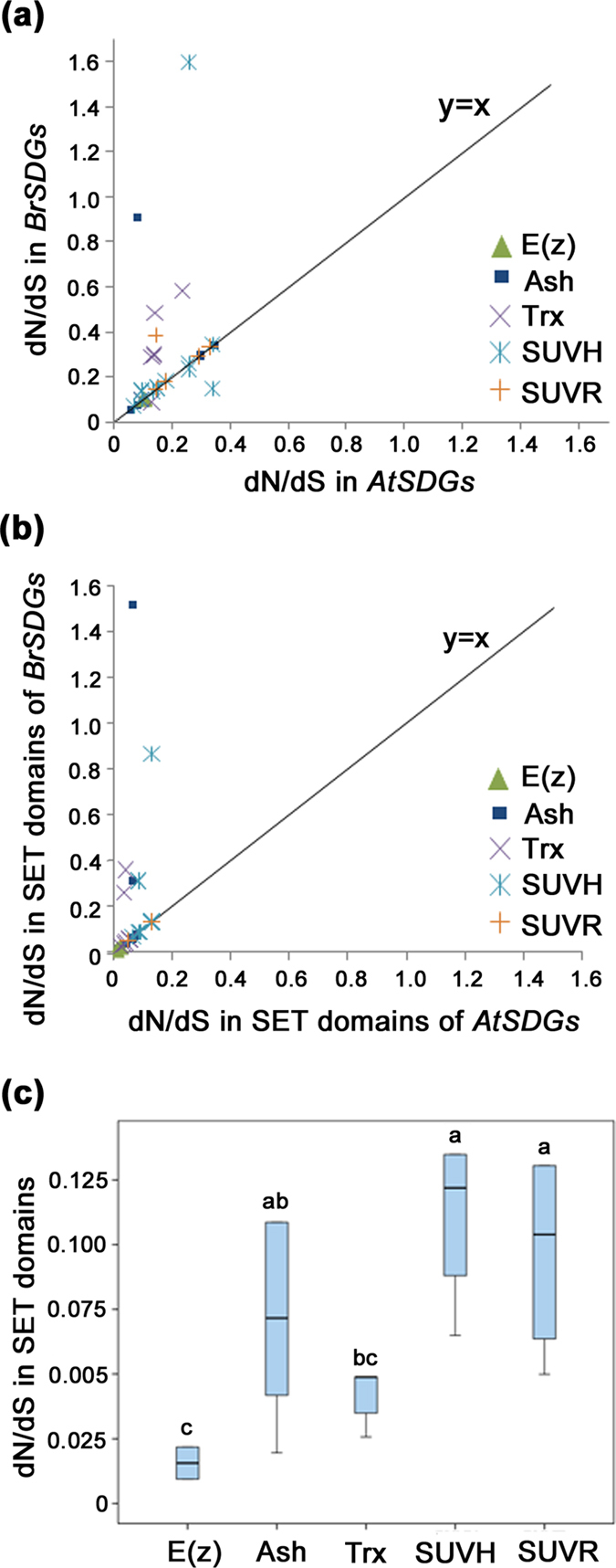
Rates of molecular evolution (dN/dS) for *SDGs* and SET domains in the four main groups of *SDGs* in *Brassica rapa* and *Arabidopsis thaliana*. (**a**) dN/dS for *SDGs*; (**b**) dN/dS for SET domains; (**c**) Average molecular evolutionary rate (dN/dS value) for SET domains in the four main groups. Different letters indicate statistical significance (*P* < 0.05) as determined by a one-way ANOVA test. The dN/dS values are in agreement with those in the corresponding acceptable models.

Because *MEA* only existed in *B. rapa* and *A. thaliana*, branch model was used to assess the molecular evolutionary rates of the other 37 *BrSDGs* from the four main groups. A one-ratio model (M0), providing a single nonsynonymous/synonymous value (dN/dS, also denoted as ω) for all branches, was used to estimate the average evolutionary rate (AER) for each gene among all studied species. AERs ranged from 0.09 to 0.27, with the mean value being 0.15 (Table S8). No significant difference was found among different groups. Further, several two-ratio and three-ratio branch models were used to constructed the acceptable model for each *BrSDGs*, and the dN/dS values of *B. rapa* branch and *A. thaliana* branch in the acceptable model were taking as the dN/dS values of *BrSDGs* and *AtSDGs* (see Methods for more detail). The dN/dS values of *BrSDGs* were more divergent than the AERs, ranging from 0.06 to 1.60. A mean value of 0.27 was also higher than the average of AERs. Compared to *A. thaliana* homologs, 11 *BrSDGs* displayed higher rates of molecular evolution, one in the Ash group, five in the Trx group, four in the SUVH subgroup, and one in the SUVR subgroup (Fig. 5a; Table S8). The overlapped symbols in Fig. 5a showed the identical rates of molecular evolution between *BrATX1* and *BrATX2*, as well as *BrSUVH1a*, *BrSUVH1b* and *BrSUVH1d*. Clearly, the Trx group contained the highest proportion (5/8) of *BrSDGs* which presented faster rates of molecular evolution (Fig. 5a; Table S8).

To determine whether the accelerated rates of molecular evolution resulted from a positive selection or a relaxed one, site models were applied to identify specific codon sites that might be under positive selection. Three pairs of models (M0/M3, M1a/M2a and M7/M8) were applied^28^. The model pairs of M0 and M3 indicated that dN/dS varied across sites (Table S9). However, the M1a/M2a and/or M7/M8 model pairs were not able to detect such specific sites (Table S9), indicating that the larger dN/dS in *BrSDGs* could be best explained by the selective constraints of relaxed selection rather than the positive one.

Subsequently, the molecular evolutionary rates of the SET domain were estimated in the same manner (Table S10). Higher rates of molecular evolution were detected in eight *BrSDGs* (Fig. 5b), with the Ash group having the largest proportion of the gene number (3/7). The mean values of AERs in the SUVH and SUVR subgroups (ω = 0.1111 and 0.0970, respectively) were significantly larger than those of the Trx and E(z) groups (ω = 0.0426 and 0.0156, respectively; *P* < 0.05). However, there was no significant difference when comparing the SUVH and SUVR subgroups to the Ash group (ω = 0.0700) (Fig. 5c).

### Expression analysis of *BrSDGs* in different tissues

Subfunctionalization always presents through different spatial expression patterns. To identify whether subfunctionalization occurred in *BrSDGs*, the expression of *BrSDGs* was tested in five tissues: roots, stems, leaves, inflorescences and siliques. No expression of *BrMEAa* and *BrSUVH1d* was detected in any of the five tissues. Also, sequence similarities and nonspecific amplification were detected for *BrSUVH7a* and *BrSUVH7c*. Therefore, in total, the expressions of 60 *BrSDGs* were analyzed.

**Figure 6:**
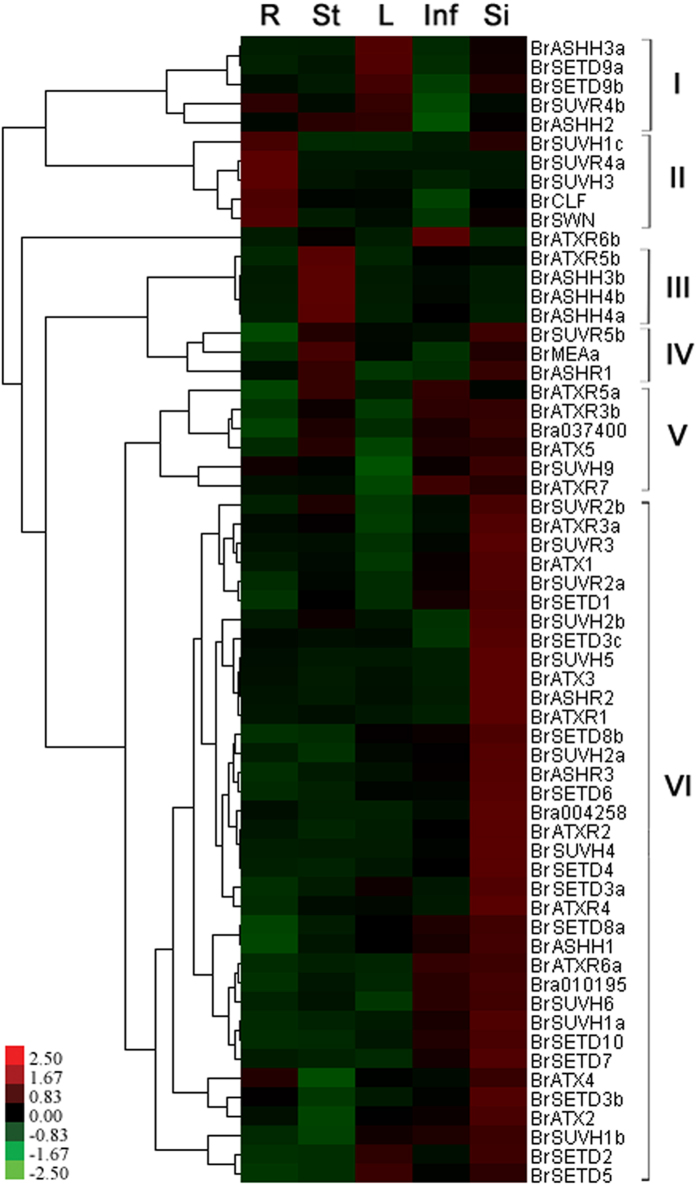
Expression patterns of *BrSDGs* in different tissues of *Brassica rapa*. qPCR was used to detect the gene expression levels in root (R), stem (St), leaf (L), inflorescence (Inf) and silique (Si).

Most *BrSDGs* were expressed in all five tissues and they were classified into six Classes in accordance with level and pattern of differential expression (Fig. 6). The genes in Class I were mainly expressed in leaves, whereas genes in Class II were largely expressed in roots, and Class III genes showed high expression levels in stems. Those in Classes IV, V and VI mostly had high expression level in siliques, although Class IV genes were also expressed in stems and Class V genes were detected in both inflorescences and stems (Fig. 6).

**Figure 7:**
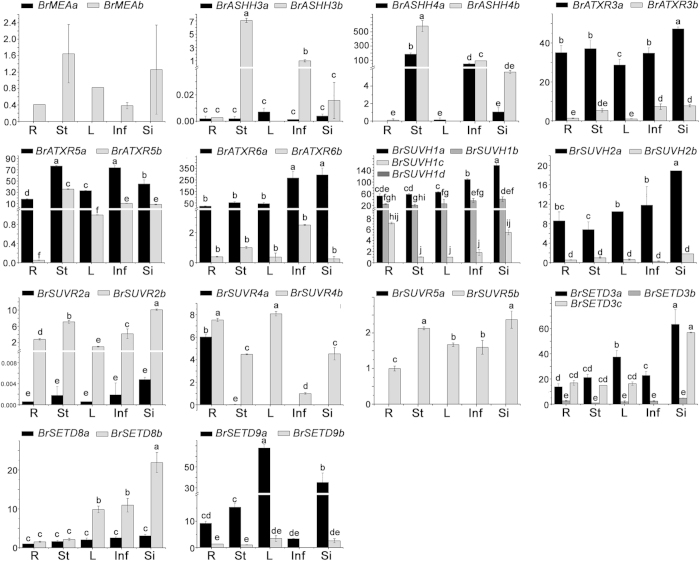
Expression patterns of each homologous *BrSDG* gene pair in different tissues of *Brassica rapa*. The △△Ct method was applied to each gene pair, and the sample with the highest Ct value smaller than 35 was chosen as the control. Different letters indicate statistical significance (*P* < 0.05) as determined by a one-way ANOVA test. The genes for which no expression was detected are listed in the figures as well. R, root; St, stem; L, leaf; Inf, inflorescence; Si, silique.

With the exception of *BrASHH4a*/*BrASHH4b* and *BrSETD9a*/*BrSETD9b*, the other duplicated *BrSDGs* (12 of 14 pairs) displayed differing expression patterns in *B. rapa*. Notably, one duplicated *BrSDGs* always showed a significantly higher expression level than the others in all tissues (Fig. 7). This situation was especially obvious in *BrASHH3a*, *BrATXR3a*, *BrATXR6a*, *BrSUVH1a*, *BrSUVH1b*, *BrSUVH2a*, *BrSUVR2b*, *BrSETD3a*, *BrSETD3c*, *BrSETD8b* and *BrSETD9a* when compared with other homologs in *B. rapa* (Fig. 7).

In order to evaluate the differences in expression patterns between *BrSDGs* and *AtSDGs*, the expressions of *AtSDGs* in *A. thaliana* were analyzed according to the microarray data in Genome Expression Omnibus database (GEO). Expression data for *AtSDGs* in inflorescences were unavailable, so the expressions of *AtSDGs* were investigated only in the following four tissues: roots before bolting, stems at 2nd internode, cauline leaves, and siliques at seed stage 3. The expression patterns of *AtSDGs* were different from those of *BrSDGs* (Fig. S7). Surprisingly, the expression of up to 27% single-copy genes of the *AtSDGs* was not detectable in the microarray.

### Group evolutionary rate analysis of the four principal groups

Few studies have compared the evolutionary rate of different groups in the *SDG* gene family. Therefore, the data of gene structure, domain architecture, subcellular localization and the rate of molecular evolution were integrated to estimate the group evolutionary rate (GER) of *BrSDGs* among the four main groups (Table 2).

The Trx group showed the highest score of GER with Trx and SUVR scoring 5.6 and 4.5, respectively. The E(z) group evolved much slower with a score of 3. The SUVH subgroup and the Ash group evolved at the slowest rates of 2 for SUVH and 1.4 for Ash.

## Discussion

Our results illustrated that the expansion of the *BrSDG* family was primarily due to the WGT event, with rearrangement and tandem duplication taking place at some loci. A lot of *BrSDGs* were lost after the WGT event. Moreover, apart from the 67 *BrSDGs*, two more *AtSDGs* are found inadvertently, increasing the number of *AtSDGs* from 47 to 49 (Fig. 1a; Table S1). Previous studies suggest that the *SDG* gene family in plants is evolutionarily conserved^29^. However, recently work on *P. trichocarpa* illustrated that when compared to *AtSDGs*, *PtSDGs* were largely retained in their number of genes and functionally diverged at the structure and expression levels^7^. In this study, comprehensive analysis performed on *SDGs* between *B. rapa* and *A. thaliana* also illustrated *BrSDGs* were divergent from *AtSDGs* at a high frequency.

The expansion of *SDG* gene family in *B. rapa* resulting from the WGT event allows nonfunctionalization, subfunctionalization and neofunctionalization^30^. Nonfunctionalization may still be an on-going process since some genes lost critical domains and/or motifs and only have a weak expression level or even no expression. Subfunctionalization is clear in *BrSDGs*, as most duplicated *BrSDGs* display different spatial expression patterns (Fig. 7). Some novel domains appeared in the *SDG* gene family for the first time, suggesting that *BrSDGs* are going through neofunctionalization (Fig. S7). Moreover, some BrSDG proteins displayed different subcellular localization patterns to their *A. thaliana* homologs, proving their potential as HKTMases for non-histone proteins, which have been detected in animals and humans^31^. This is another line of evidence for neofunctionalization in *BrSDGs*.

*B. rapa* is widely cultivated throughout the world with many subspecies, varieties and variant types. These vegetable crops demonstrate significant morphological diversity with various types, such as root vegetables, stem vegetables and leafy vegetables, and also display a broad diversity in growth habit^32^. The genomic rearrangement and gene-level evolution after the common WGT event in the *Brassica* genus has contributed to the rich variety of morphotypes in *Brassica* species^29^,^33^. It has been further suggested that the auxin-related genes and genes involved in flowering time control, propelled the expansion of the rich variety of morphotypes^20^,^29^. As an important epigenetic regulatory gene family, several *SDGs* (*AtATXR3*, *AtASHH2* and *AtATX1*) are involved in regulating auxin-related genes^34^,^35^. All flowering time genes, except *CONSTANS* (*CO*), are *CLF* target loci^8^, while a series of *SDGs* targeting H3K4 and H3K36 residues regulate *FLC* in *A. thaliana*^11^,^12^,^13^,^14^. Moreover, *SDGs* also play a role in shaping other aspects of the morphotype, such as shoot branching, leaf size, root length and the number of lateral roots^36^,^37^. In conclusion, the *SDG* gene family displays high divergence in *B. rapa*, which may have contributed to the rich variety of morphotypes in the *Brassica* genus, though this has to be investigated further by analyzing the *SDG* family in each of the varieties.

In the long process of evolution, selection pressure is powerful in shaping gene families, resulting in different evolutionary patterns among gene families and even different groups in one gene family^38^,^39^. The *SDGs* in the E(z) and Trx groups are believed to be more conserved than those in the Ash and Suv groups^29^, as the E(z) and Trx proteins are restricted to methylate a single histone residue while the Ash and Suv proteins target more residues^29^,^40^. This is consistent with our analysis on the rate of molecular evolution of the SET domain, in which the mean values of dN/dS in E(z) and Trx are smaller than those in SUVH, SUVR and Ash (Fig. 6c).

Our study also demonstrates that the group evolutionary pattern of the whole set of genes is totally different from that of SET domain. The analysis on gene structure and motif architecture illustrated that the SET domain is relatively conserved. Thus, it is proposed the wide difference in group evolutionary pattern displayed by the whole gene set and the SET domain is due to the regions outside the SET domain, which are necessary for recognizing and binding biomacromolecules and leading the HKMTases to specific target loci (Tables S5 and S6). Two reasons are suggested to account for this pattern. Firstly, *B. rapa* suffered extensive chromosome rearrangement and genome shuffling^41^, and we hypothesis that during the evolution of *SDG* genes, they are forced to passively change their recognition and binding regions in order to adapt to the changes of the genome. As for the unique conservative property in different parts of the genome, the *SDG* gene family may have different group evolutionary rates. Evidence shows that the E(z) and Trx proteins are always recruited to promoters^40^, while the Ash group proteins are enriched at the transcription regions^42^ and Suv proteins are regulators of transposon chromatin^43^. That is to say, the promoter is less conserved than the transcription region and the fixed transposable sequences which are abundant in centromeric and pericentromeric heterochromatin. This lends good support to our above hypothesis.

**Figure 8:**
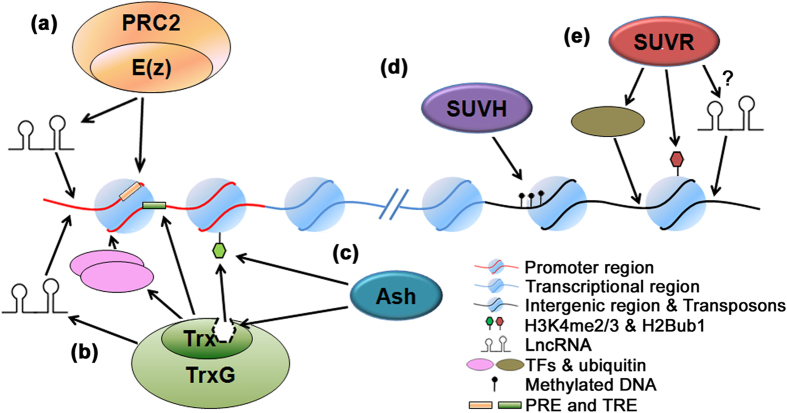
SDG proteins in different groups are recruited to their target loci through different mechanisms. (**a**) E(z) proteins interact with lncRNAs or DNA sequences called PcG response elements (PREs); (**b**) Ash proteins are recruited by histone modifications or bound directly to Trx proteins; (**c**) Trx proteins can be recruited to the target locus through various methods, such as binding to TREs (Trx response elements), interacting with lncRNAs, recognizing other histone modifications, linking to transcription factors (TFs) or polymerase-associated factor 1 (PAF1); (**d**) SUVH proteins recognize different types of methylated DNA; (**e**) SUVR proteins target ubiquitin, H2Bub1 and perhaps lncRNAs.

All groups of the SDGs have a different capacity in recognizing and binding with biomacromolecules, therefore, we hypothesize those who can recognize more types of biomacromolecules could have been evolved faster. E(z) proteins can be recruited by DNA sequences called Polycomb Repressive Complex2 (PRC2) response elements (PREs) and long noncoding RNAs (lncRNAs)^44^,^45^ (Fig. 8a). The Trx proteins can recognize proteins [transcription factors (TFs) or polymerase-associated factor 1 (PAF1)], lncRNAs, other histone modifications, and DNA sequences called Trx response elements (TREs)^46^ (Fig. 8b). The Ash group proteins bind to methylated H3K4^47^ or interact directly with Trx or other Ash proteins^48^ (Fig. 8c), while the domains in the SUVH subgroup only enable them to bind with methylated DNAs (CG, CHG, CHH)^43^ (Fig. 8d). In conclusion, the Trx proteins target the less conserved promoter regions, participate in complex transcription initiation processes, and recognize various types of biomacromolecules, leading to the fastest GER. The E(z) group proteins also target promoter regions but having fewer mechanisms for recognizing target loci, limits its potential to evolve faster. The target loci and recognition sites for the Ash and SUVH proteins are much simpler, resulting in a much slower evolutionary rate. Finally, as for the SUVR subgroup, it has the ability of binding with ubiquitin and mono-ubiquitinated histone H2B (H2Bub1)^49^. In addition, two novel domains, Ribosomal_L14 and Stress-antifung, indicate its potential in recognizing other biomacromolecules, such as lncRNAs. Moreover, SUVH proteins are involved in H3K9me1/2 in heterochromatin, while the question of which HKMTases are responsible for the high level of H3K9me3 in euchromatin is still unknown^39^. AtSUVR4 was the first discovered H3K9me3 methyltransferase in plants^49^ and although restricted to methylate transposon chromatin, other members in this subgroup could potentially regulate the H3K9me3 in euchromatin. And this explains why the SUVR subgroup has a relatively higher GER, which ranges between the Trx and the E(z) group.

## Methods

### Plant materials and growth conditions

Chinese cabbage (*Brassica rapa* ssp. *chinensis*) ‘Aijiaohuang’ were planted in an experimental greenhouse at Zhejiang University. Roots, stems, leaves and inflorescences were collected during the flowering stage (22 weeks after sowing). Germinal siliques were harvested 48 hours after artificial pollination. Tobacco plants (*Nicotiana benthamiana*) were grown in soil in a growth chamber under a 24/22 °C day/night temperature and a 16/8 h photoperiod. Six-week-old plants were used for transient expression analyses of the *SDGs*.

### Identification of *SDGs*

The genomic and predicted proteomic sequences of *B. rapa* were retrieved from the *Brassica* Database (BRAD) (ver. 1.5, http://brassicadb.org/brad/index.php). To identify the genes containing a SET domain in *B. rapa*, the SET domain PF00856 from the Pfam database (http://pfam.sanger.ac.uk/) was used to search the BRAD. For the loci containing repeat tandem genes, DNA sequences were used for protein prediction in FGENESH (http://linux1.softberry.com/berry.phtml?group=programs&subgroup=gfind&topic=fgenesh). For all candidate genes, both the Simple Modular Architecture Research Tool (SMART) (http://smart.embl-heidelberg.de/smart/change_mode.pl) and Pfam were used to confirm their SET domains.

Sequences of *AtSDGs* were collected from The *Arabidopsis* Information Resource (TAIR) (ver. 10, http://www.arabidopsis.org/). *SDGs* in other species such as *O. sativa*, *P. trichocarpa*, *S. moellendorffii*, *P. patens*, *C. reinhardtii* and *V. carteri* were gathered according to previous work^29^ from the Joint Genome Institute database (JGI) (http://genome.jgi.doe.gov/).

### Phylogenetic analysis

The SET domains used for phylogenetic analysis were obtained from full-length SDG amino acid sequences according to the prediction from Pfam. Multiple-sequence alignment was performed using MUSCLE with standard settings and some manual alignment in MEGA6 software^50^. MEGA6 was further applied for the construction of a phylogenetic tree using the neighbor-joining (NJ) method with 1,000 bootstraps.

### Syntenic and retention proportion analysis

The distribution for the 24 building blocks of the ancestral karyotype (AK) was carried out according to a previous study^51^. The chromosomal locations of *BrSDGs* and *AtSDGs* were obtained from BRAD and TAIR, respectively, and used to place the genes within syntenic blocks. The syntenic relationships between or within the genomes were illustrated using Circos^52^.

For the retention proportion analysis, the neighbor genes were defined as 10 genes on each flanking side of the *AtSDGs*. A set of 458 core eukaryotic genes and 458 random genes were downloaded from CEGMA (http://korflab.ucdavis.edu/Datasets/cegma/#SCT4) and used to search for the *Brassica* syntenic genes in BRAD.

### Gene structure and domain architecture analyses

The gene structures of *SDGs* in the same clade were phylogenetically analyzed. DNA sequences were filtered from BRAD and the intron phases were analyzed using the Gene Structure Display Server (GSDS) (http://gsds.cbi.pku.edu.cn/) with some manual adjustment. If the intron located behind the third nucleotide of a codon, it is defined as Phase 0; if the intron located between the first and second nucleotides of a codon, it is defined as Phase 1; and the introns located between the second and third nucleotide of a codon are Phase 2^53^.

Both SMART and Pfam were used to retrieve the full-length amino acid sequences for building domain architecture. MEME (http://meme.nbcr.net/meme/) was used to identify the short conserved motifs among the SET domains.

### Subcellular localization of SDG proteins

The analysis of the subcellular localizations of BrSDGs and AtSDGs were predicted using bioinformatics methods NucPred^54^ and WoLF PSORT^55^. The threshold scores were set at 0.5 for NucPred and 7 for WoLF PSORT (*knn* = 14)^56^.

Full-length coding sequences of candidate *SDGs* were amplified (Table S11) and cloned into the *pFGC-EGFP* vector. Tobacco leaf epidermal cells were infiltrated with cultures (OD_600_ = 0.4) of transformed *Agrobacterium tumefaciens*. Confocal imaging was performed 24 h after infiltration using an inverted Zeiss LSM 510 META CLSM (Jena, Germany) with an Argon laser (488 nm) and a 488–511 nm band pass filter. Images were analyzed using an LSM 5 Image Browser (Jena, Germany) and Photoshop 7.0 software.

### Calculation of the rate of molecular evolution

The rate of molecular evolution for each *SDG* in the four main groups was estimated by the dN/dS value using the CODEML program in PAML 4.7^57^. Full SDG protein sequences from *B. rapa*, *A. thaliana*, *O. sativa*, *P. trichocarpa*, *S. moellendorffii* and *P. patens* were used to construct the guide trees using the NJ method in MEGA6 (Fig. S8).

M0 was used to estimate AER (Fig. S9a) and a two-ratio branch model (M-br), which allowed the dN/dS value to vary between the branch for *B. rapa* and other species, was employed to estimate the evolutionary rate of *BrSDGs* (Fig. S9b). M-br was compared with M0 using the likelihood-ratio test (LRT). For those *SDGs* that M-br displayed no significant difference from M0, M0 was chosen as the acceptable model. For the other *SDGs*, a similar model named M-at (Fig. S9c), which allowed the dN/dS value to vary between the branch of *A. thaliana* and others, was further calculated. If a difference also existed between M-at and M0, it was speculated the larger dN/dS in *BrSDGs* was due to the accelerated evolution in the common branch of *B. rapa*/*A. thaliana* cluster or the *B. rapa*. Thus, a two-ratio model (M-ab) (Fig. S9d) was constructed to allow a different dN/dS value to exist between the common branch of the *B. rapa*/*A. thaliana* cluster and other branches. A three-ratio model (M-ab-br) (Fig. S9e) was compared with the M-ab model using LRTs for choosing the acceptable model. And the difference in the rates of molecular evolution between *BrSDGs* and their *A. thaliana* homologs was defined by calculating the dN/dS value in the *B. rapa* branch and the *A. thaliana* branch under the acceptable model. The rates of molecular evolution in the SET domain were also estimated using the branch model and guide trees that were used for *SDGs*.

A site model was further implemented to detect specific sites under positive selection in the faster evolution of *SDGs*^28^. Three pairs of models (M0/M3, M1a/M2a and M7/M8) were applied, and a Bayes empirical Bayes (BEB) approach was used to identify specific amino acids subjected to positive selection^57^.

### Expression analysis

qPCR was used to detect the expression of *BrSDGs* in different tissues of Chinese cabbage. *BrUBC10* was used as the reference gene and qRT-PCR was carried out in triplicate using gene specific primers (Table S11) according to previous study^58^. Agarose gel electrophoresis was used to confirm that only a single, specific PCR product was amplified. The PCR products of highly homologous fragments were cloned into T-vectors and sequenced to ensure specificity. For those genes where no expression was detected in any tissue, DNA fragments were cloned and subsequently sequenced to verify the primers and PCR systems used in PCR amplification.

The results were calculated using the 2^−△△Ct^ method^58^ and further gene-wise normalized, mean-centered and clustered hierarchically using the average linkage clustering method in Cluster 3.0 (http://bonsai.hgc.jp/~mdehoon/software/cluster/index.html).

The expression data for *AtSDGs* was collected from the GEO (www.ncbi.nlm.nih.gov/geo/) under accession numbers GSE5630, GSE5631, GSE5633 and GSE5634. The database did not contain information that directly corresponded with the tissues and stages for expression of *BrSDGs*, so the data for roots before bolting, stems at the 2nd internode, cauline leaves and siliques at seeds stage 3 were used. The microarray data was log-transformed and mean-centered, and then, the data were clustered in the same manner as the data for *BrSDGs*.

### Analysis of evolutionary rate among the four main groups of *SDGs*

The variety of gene structures often reflects the evolutionary potential, but does not provided substantial changes, so we assigned every changing site in gene structure a weight of 1. Protein domains are basic functional modules and subcellular localization directly determines the characteristic of a protein. Therefore, the changes in domain and subcellular localization were both weighted as 2. Moreover, the rate of molecular evolution was defined the fragments that existed in all sequences, making it also suitable to be assigned a weight of 1. Accordingly, the following equation was used to calculate the GERs:


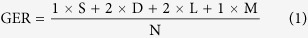


S = Number of sites with Structure changed;

D = Number of sites with Domain changed;

L = Number of genes with subcellular Localization changed;

M = Number of genes with higher rate of Molecular evolution;

N = Total gene Number in a given group.

## Additional Information

**How to cite this article**: Dong, H. *et al.* Diversification and evolution of the *SDG* gene family in *Brassica rapa* after the whole genome triplication. *Sci. Rep.* **5**, 16851; doi: 10.1038/srep16851 (2015).

## Supplementary Material

Supplementary Dataset 1

Supplementary Dataset 2

## Figures and Tables

**Table 1:** The number of *BrSDGs* with changes in gene structure compared with their homologs in *A. thaliana.*

Group	Structure changedgene number/Groupgene number	Intron-changedgene number		Exon-changedgene number		Phase-changedgene number
		Gain	Loss	Gain	Loss	
E(z)	3/4	2	0	0	2	1
Ash	3/7	0	2	0	1	1
Trx	8/8	2	5	0	4	4
ATXR5/6	3/4	0	1	1	2	0
SUVH	7/14	7	0	0	0	0
SUVR	2/6	0	1	2	0	0
SMYD	2/6	1	1	0	1	0
SETD	11/15	3	3	1	4	3
Total	39/64	15	13	4	14	9

**Table 2:** Group evolutionary rates (GERs) of the four main group of *BrSDGs.*

Group(gene number)	gene-structurechanged site number	domain-architecturechanged site number	subcellular localizationchanged gene number	larger dN/dSgene number	GER
E(z) (4)	6	3	0	0	3
Ash (7)	5	1	1	1	1.4
Trx (8)	20	10	0	5	5.6
SUVH (14)	10	6	1	4	2
SUVR (6)	10	7	1	1	4.5
