# Supplementary material for: Diversification and evolution of the SDG gene family in Brassica rapa after the whole genome triplication
Source: Sci Rep. 2015 Nov 24;5:16851. doi: 10.1038/srep16851 (PMC4657036; doi:10.1038/srep16851)
Supplement: Supplementary Dataset 1 [file srep16851-s1.doc]

**Supporting Information**

**Diversification and evolution of the *SDG* gene family in *Brassica rapa* after the whole genome triplication**

Heng Dong, Dandan Liu, Tianyu Han, Yuxue Zhao, Ji Sun, Sue Lin, Jiashu Cao, Zhonghua Chen, Li Huang

**Figure S1** Gene structure of *SDGs* in *Brassica rapa* and *Arabidopsis thaliana*.

**
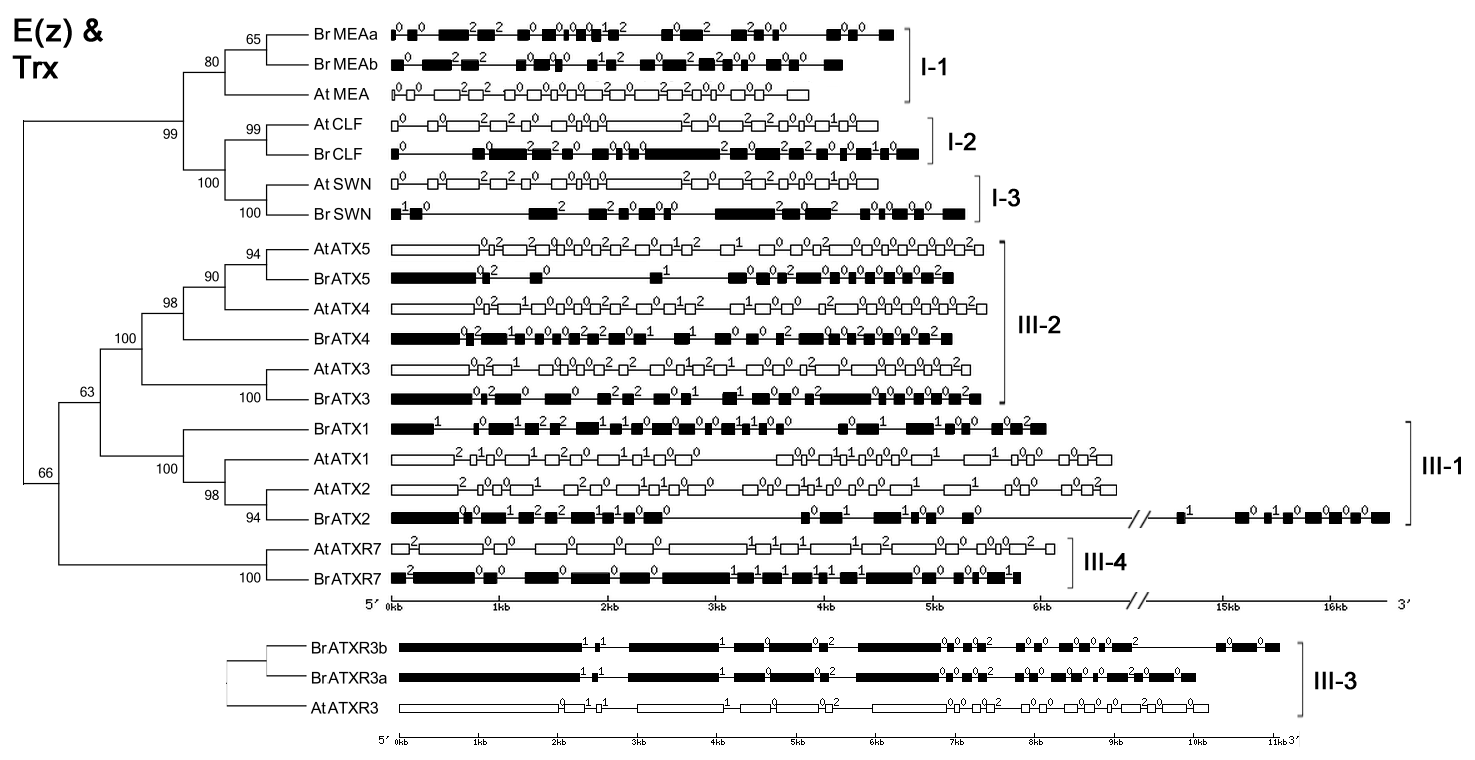

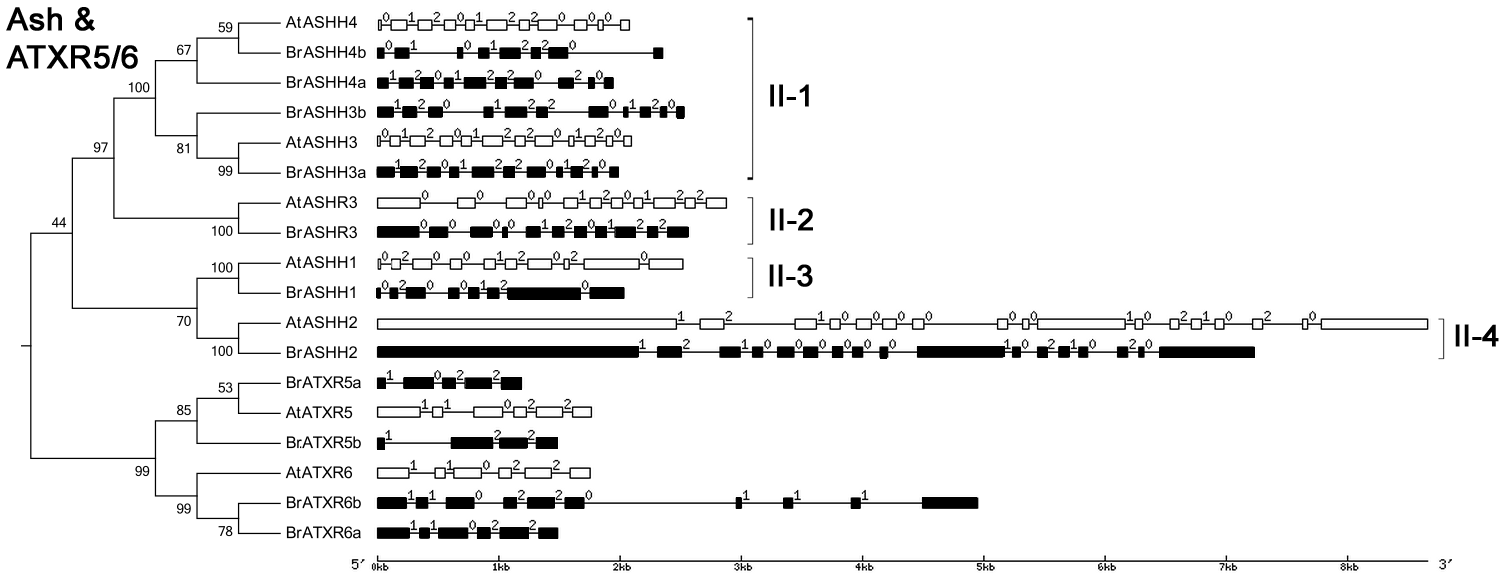
**

**
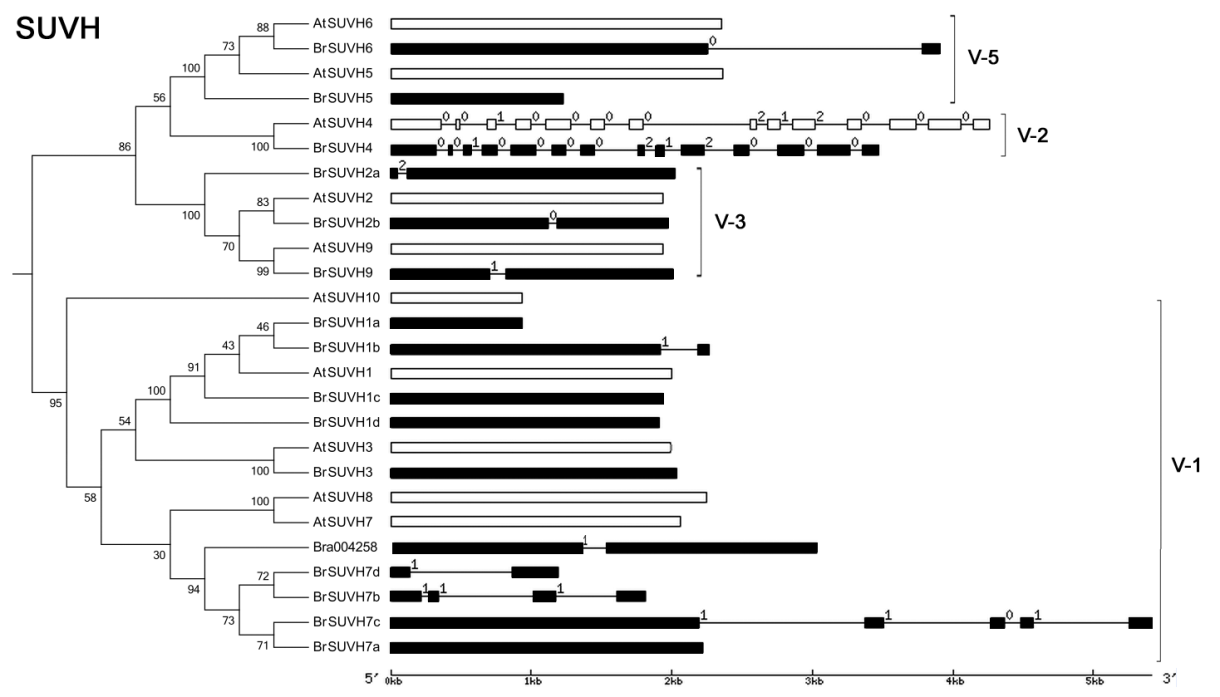
**

**
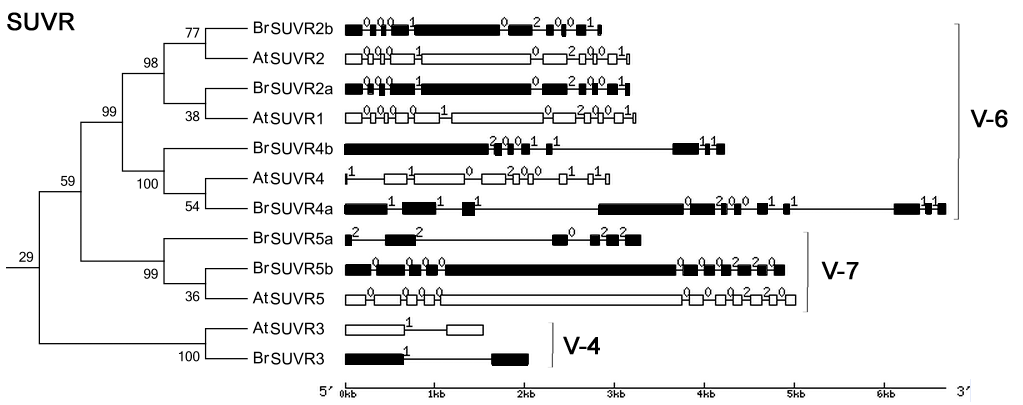
**

**
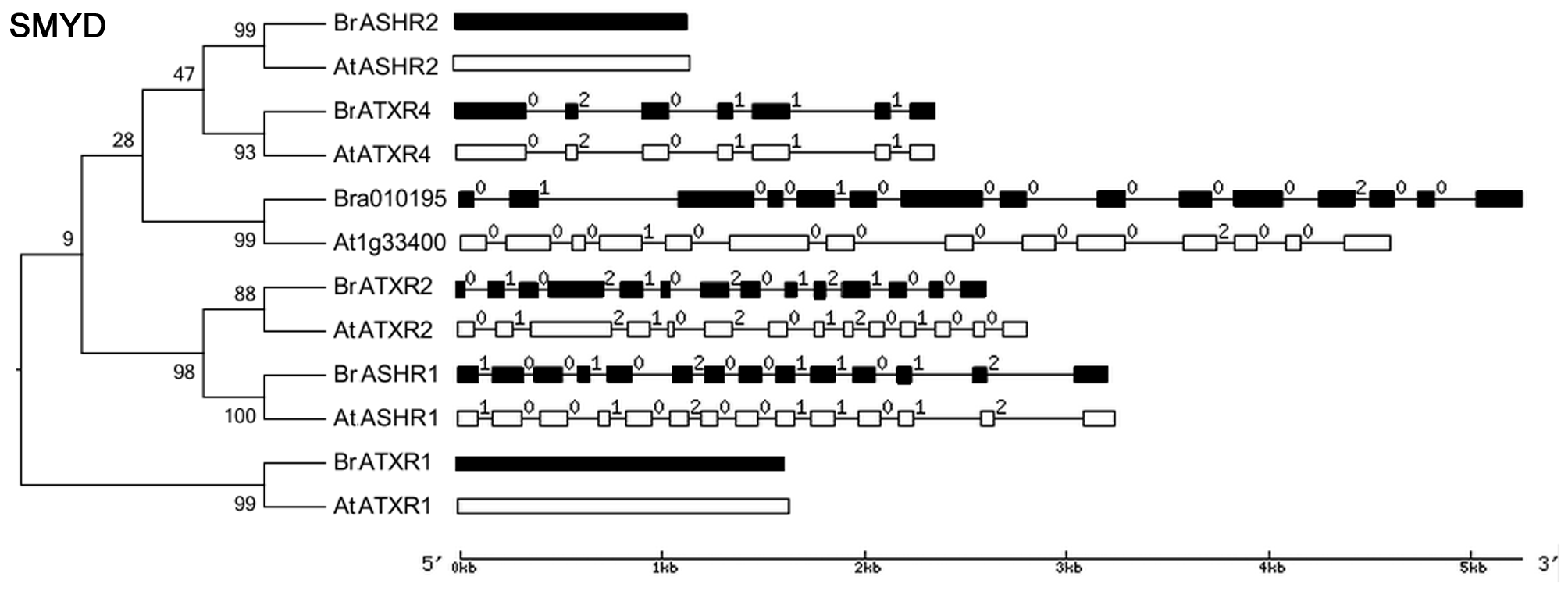
**

**
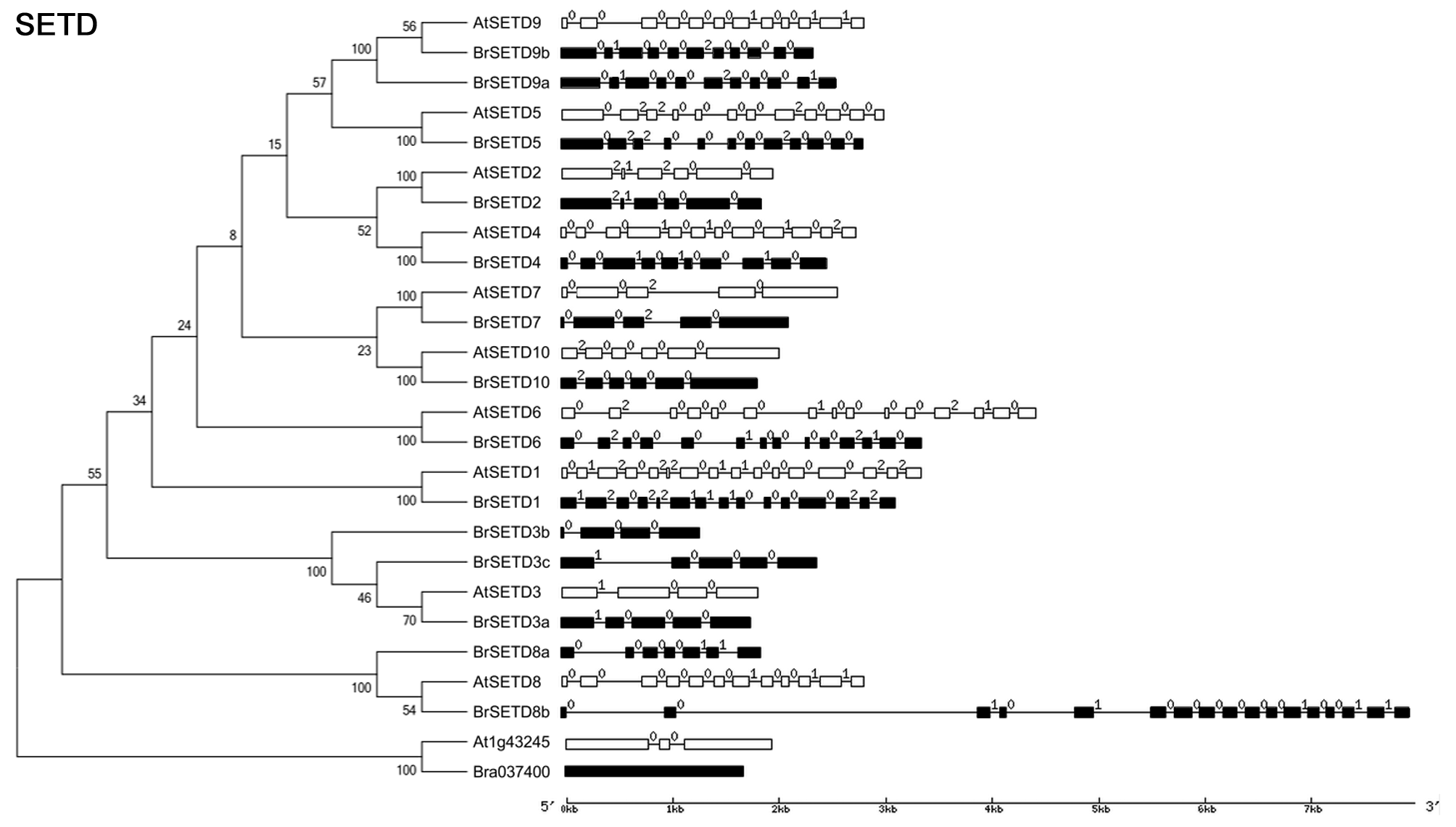
**

**Figure S2** Gene structure of *SDG* homologs in the Ash, Trx and Suv groups in selected species.


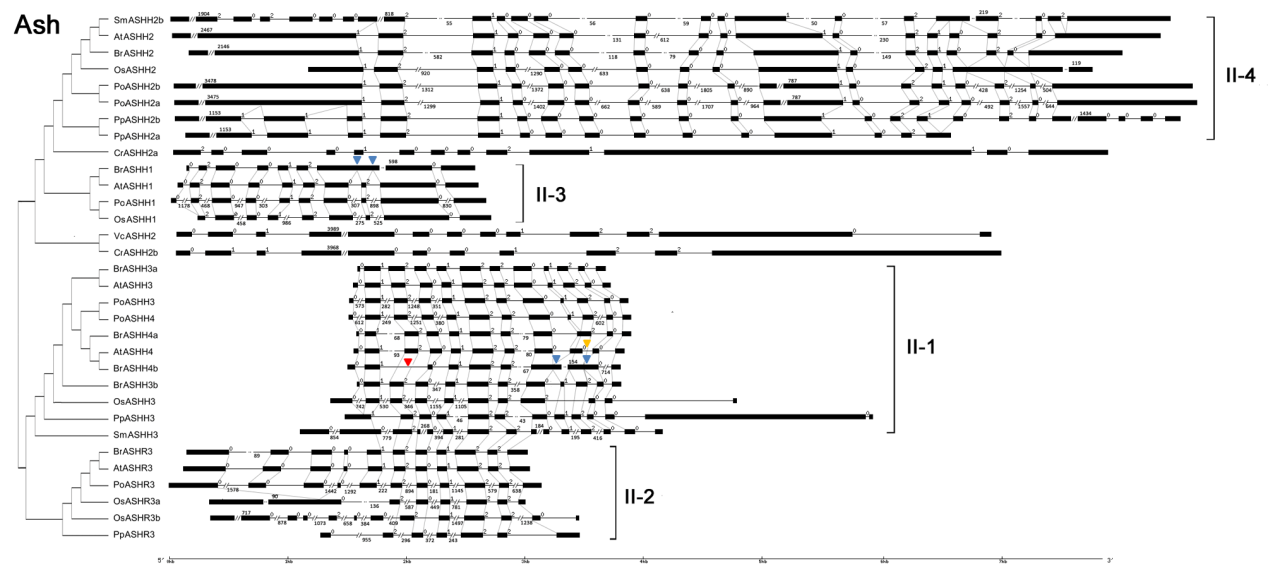


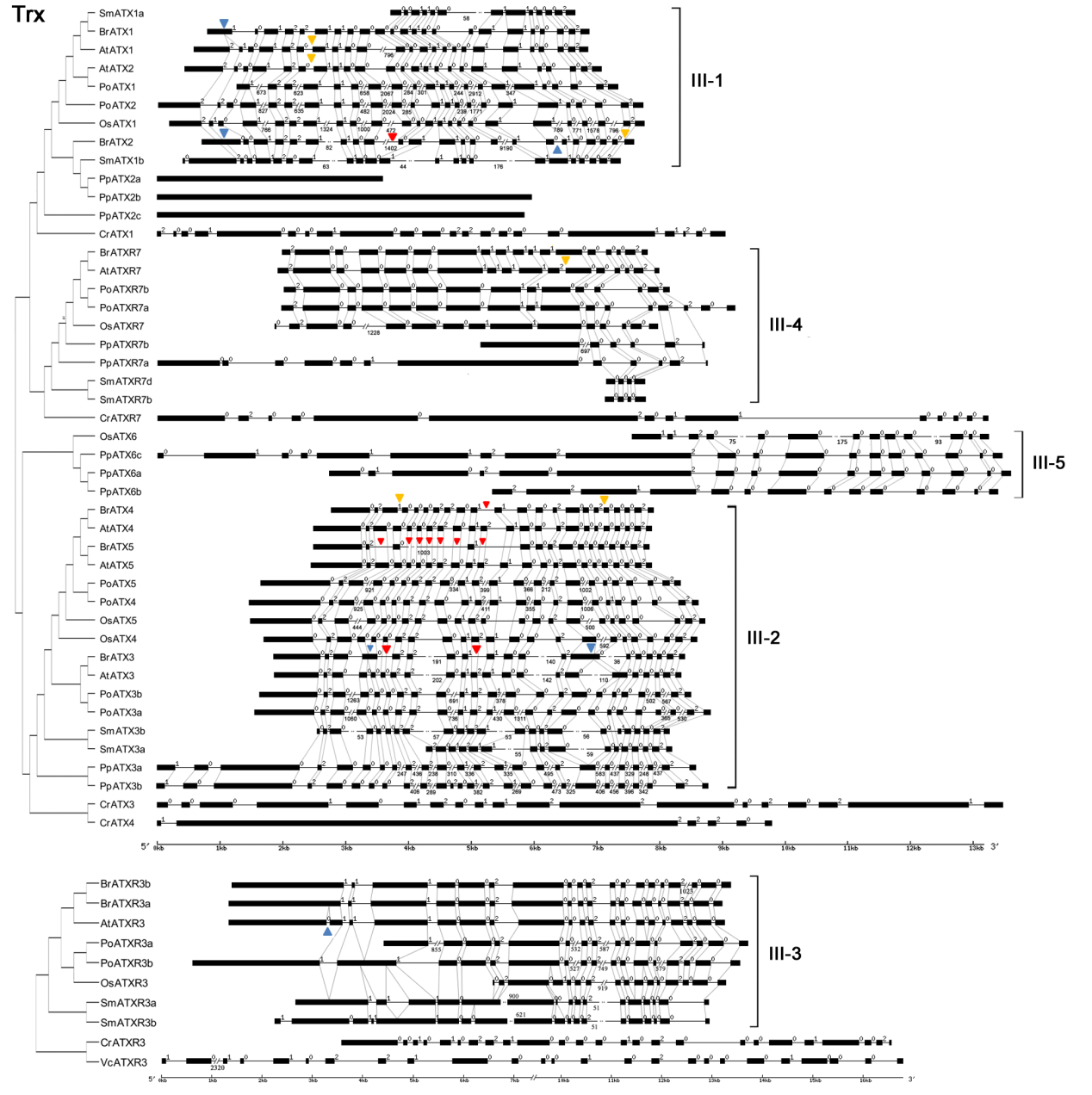


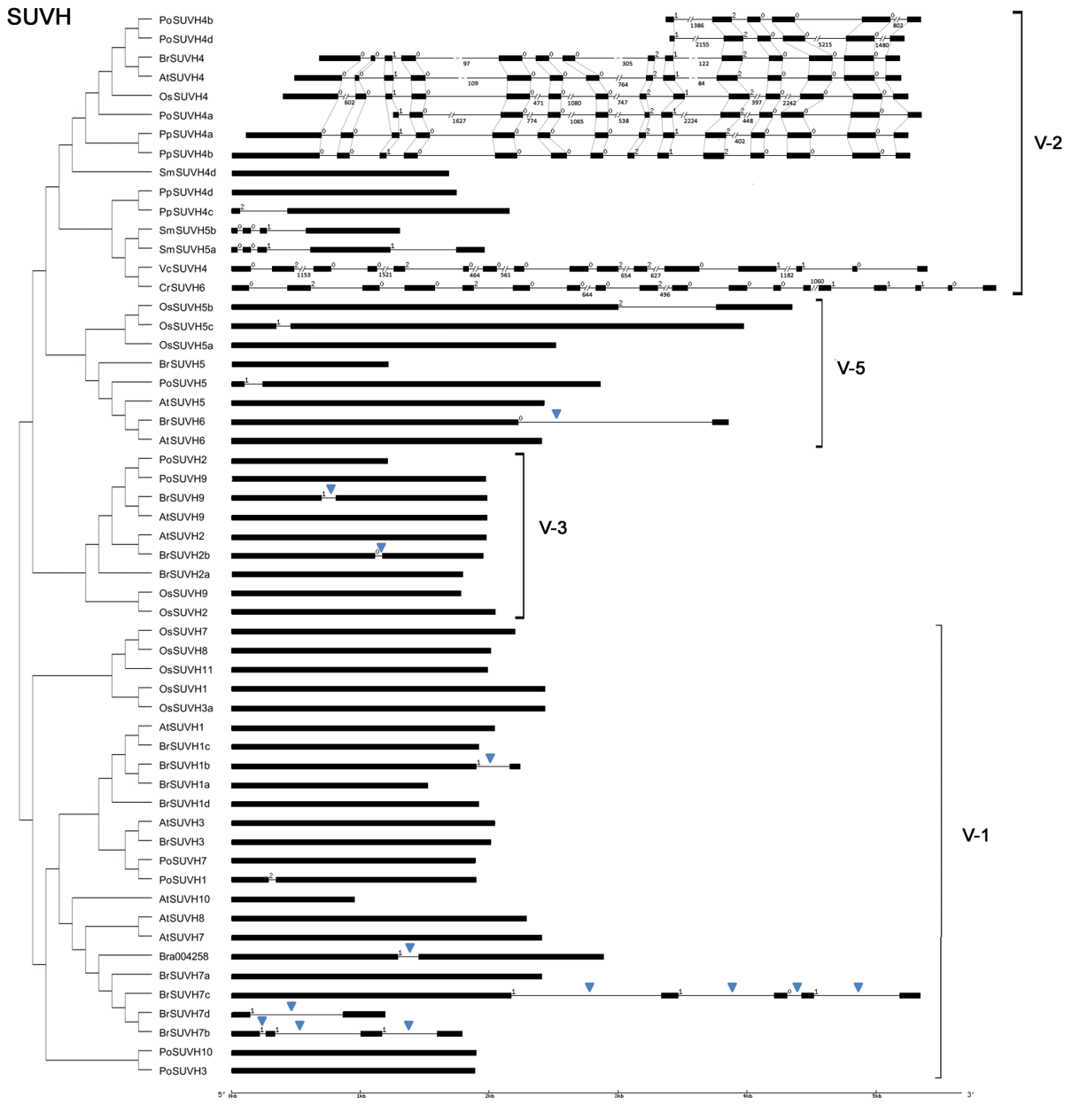

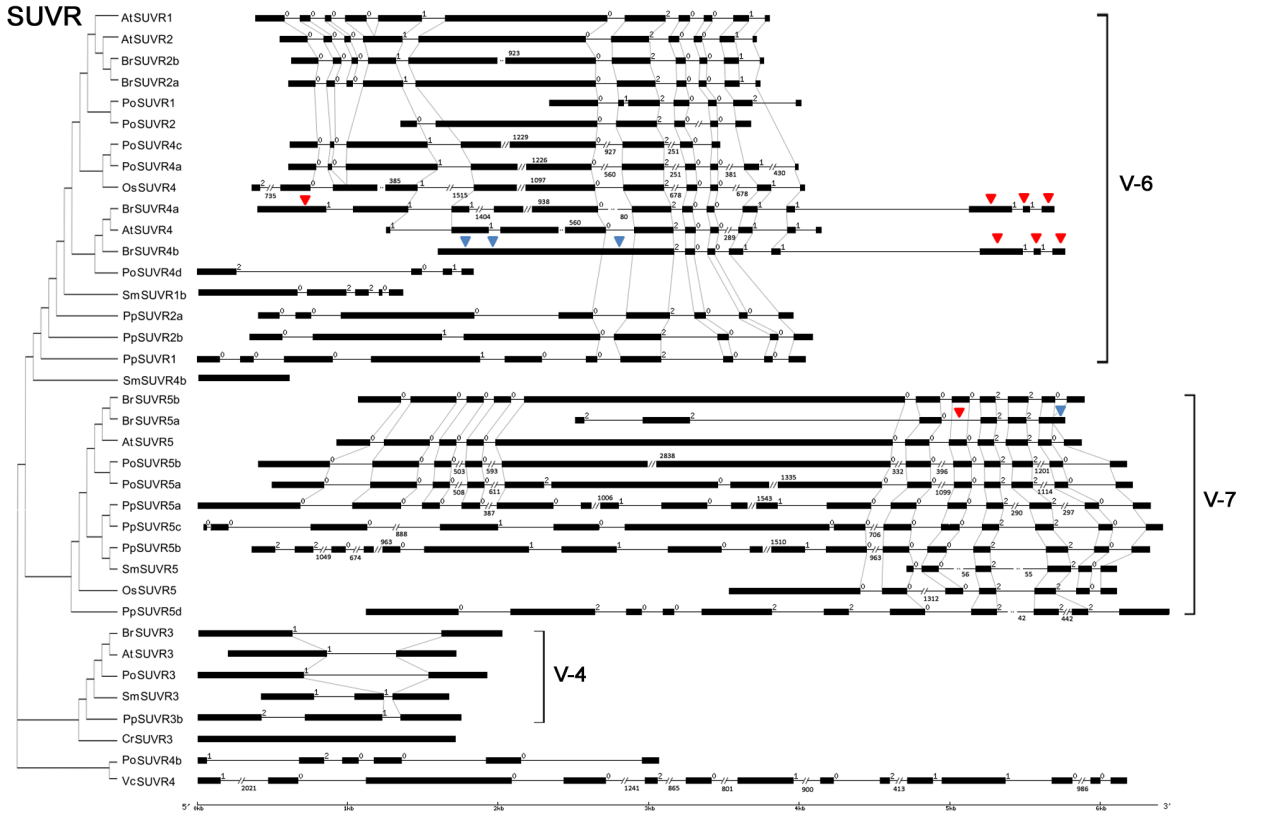


**Figure S3** Differences in domain architecture between the homologous *SDGs* in *Brassica rapa* and *Arabidopsis thaliana*.

**
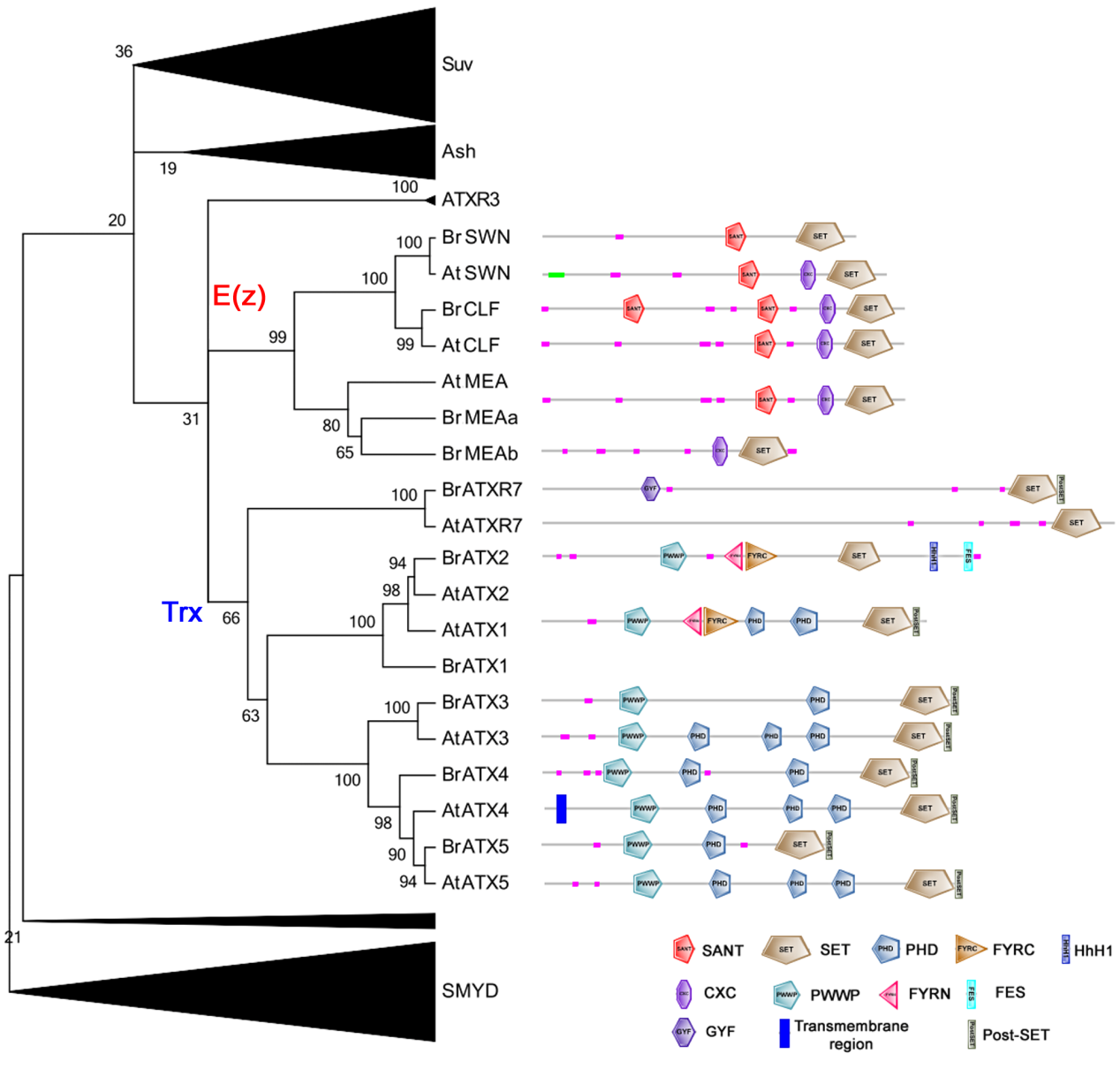
**

**
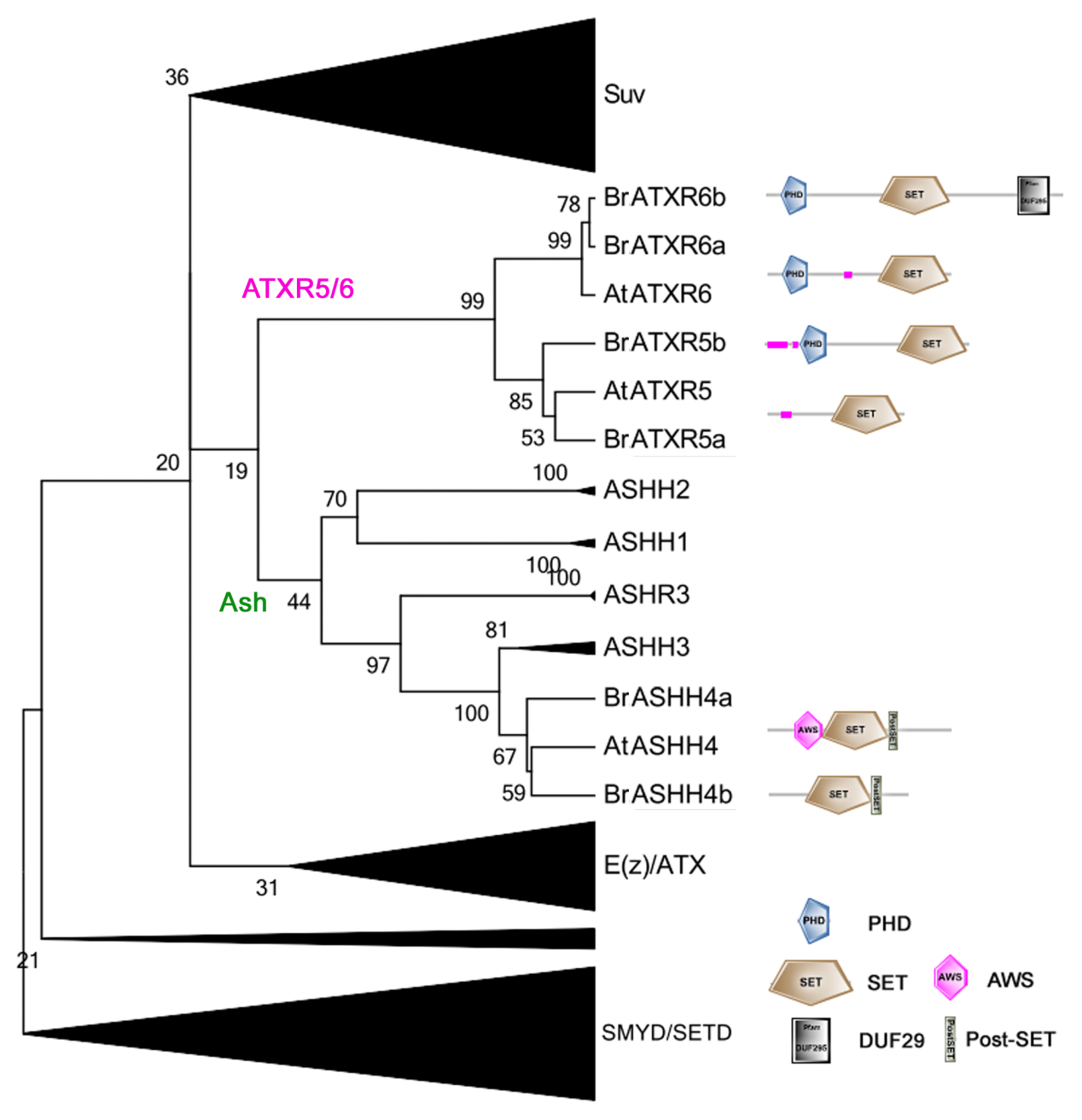

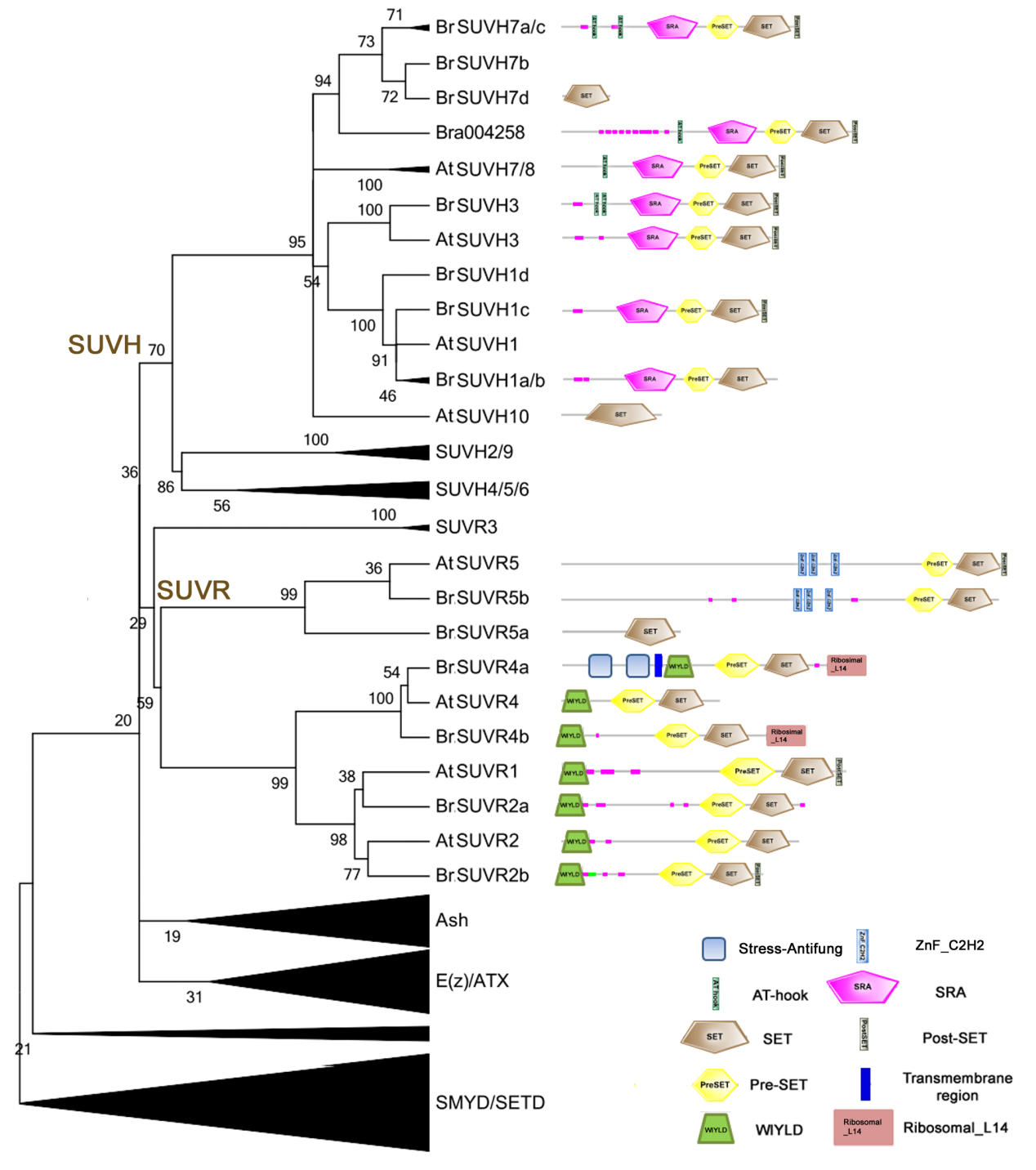
**

**Figure S4** Domain architecture of some *SDGs* in selected species.


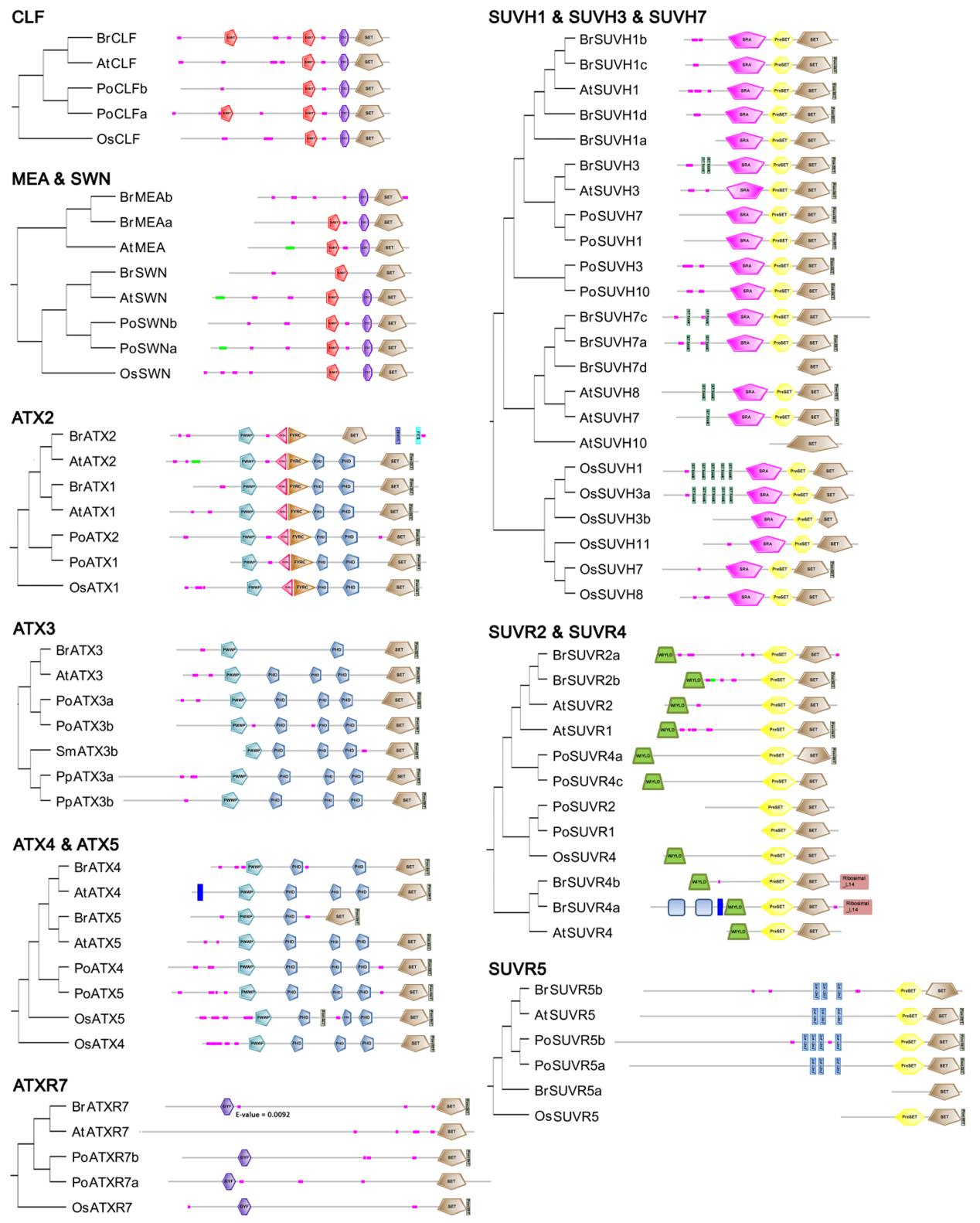


**Figure S5** MEME motif architecture of SET domains from the four main groups of *SDGs* in *Brassica rapa* and *Arabidopsis thaliana*.


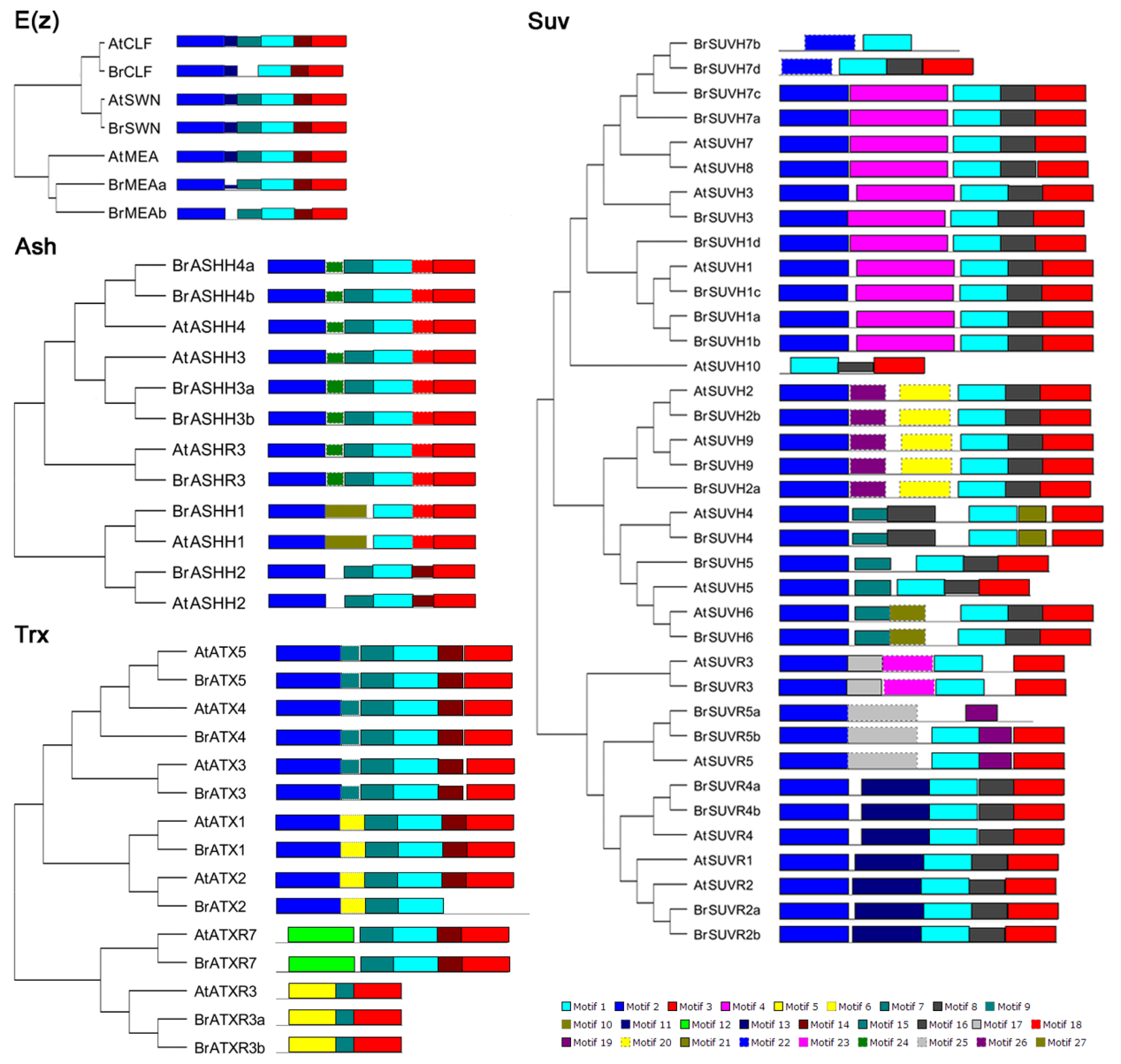


**Figure S6** MEME motifs in the SET domain.


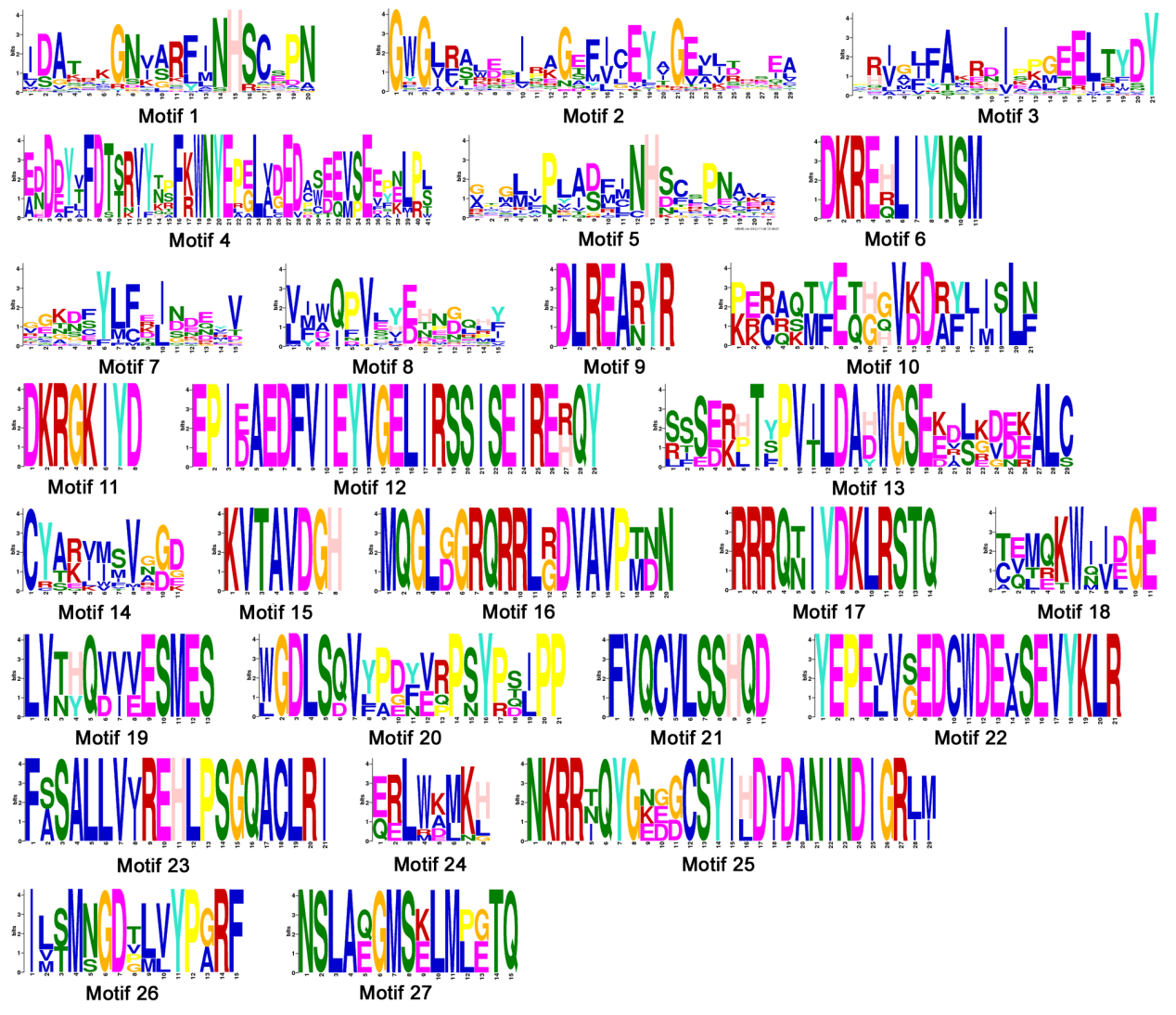


**Figure S7** Expression patterns of *SDGs* in different tissues of *Arabidopsis thaliana*.


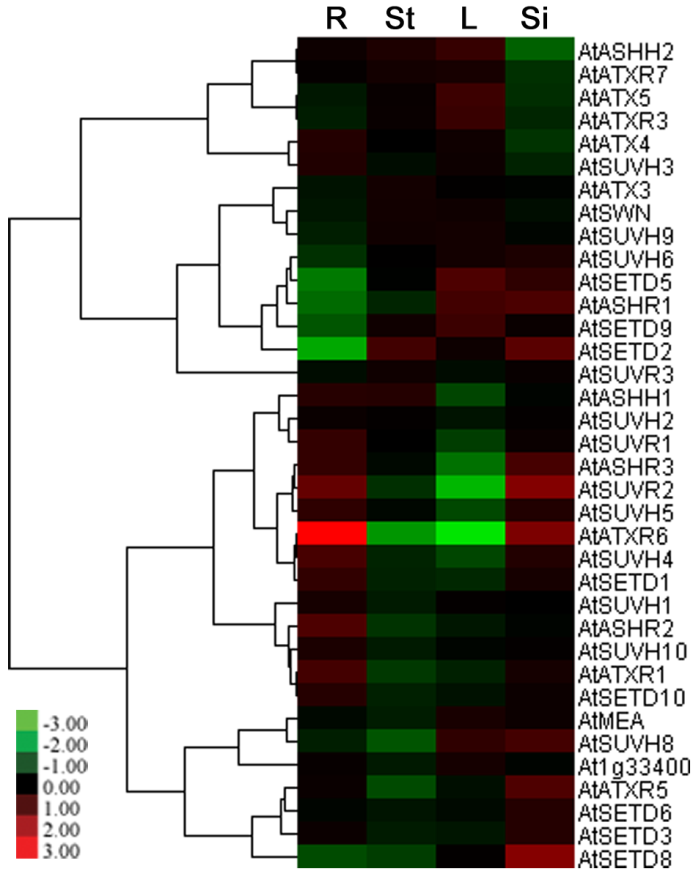


**Figure S8** Guide trees used for calculating the molecular evolutionary rate for the four main groups of *SDGs*.


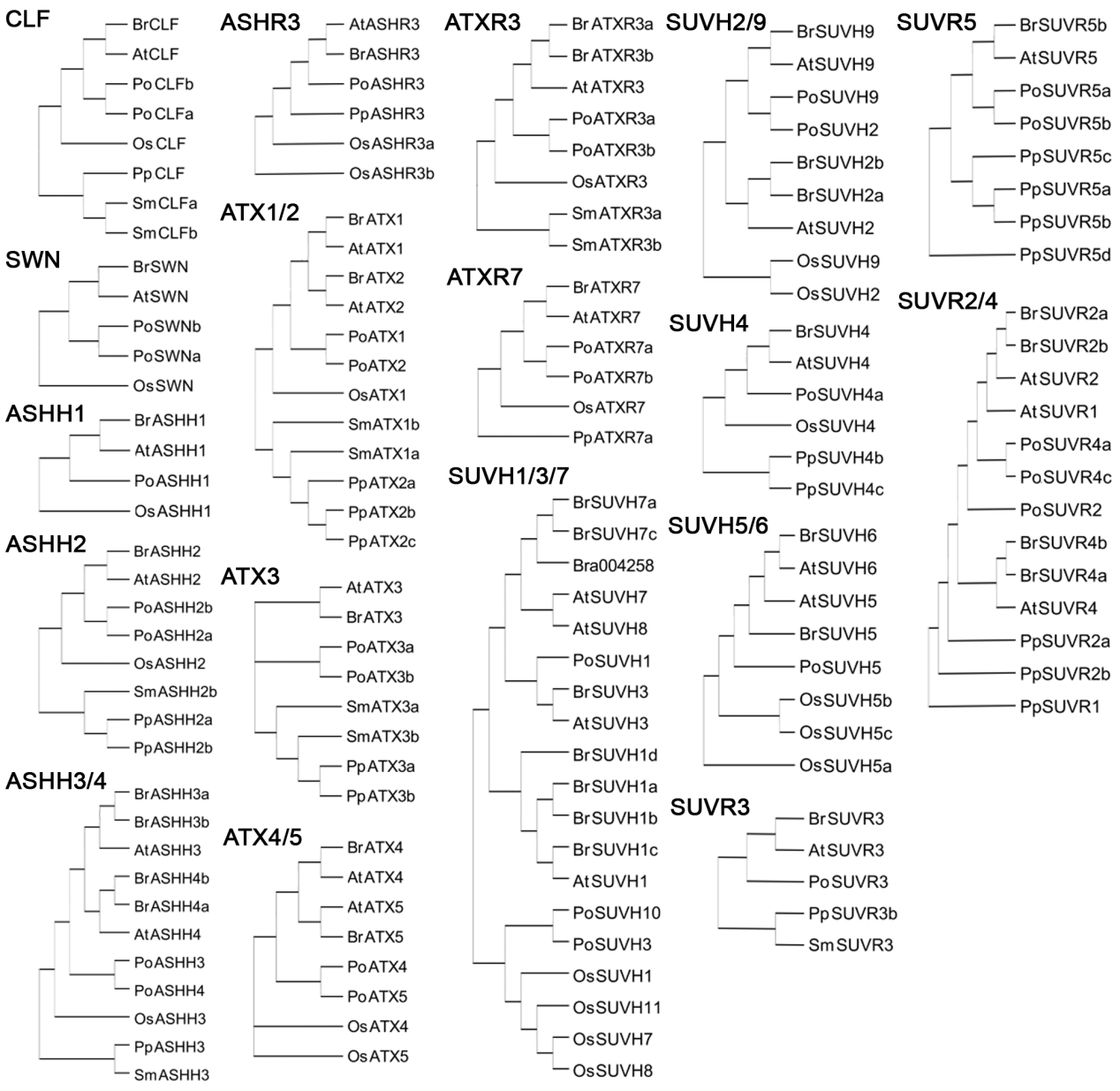


**Figure S9** Schematic of the method used for molecular evolutionary rate analysis on *BrSDGs*.

**
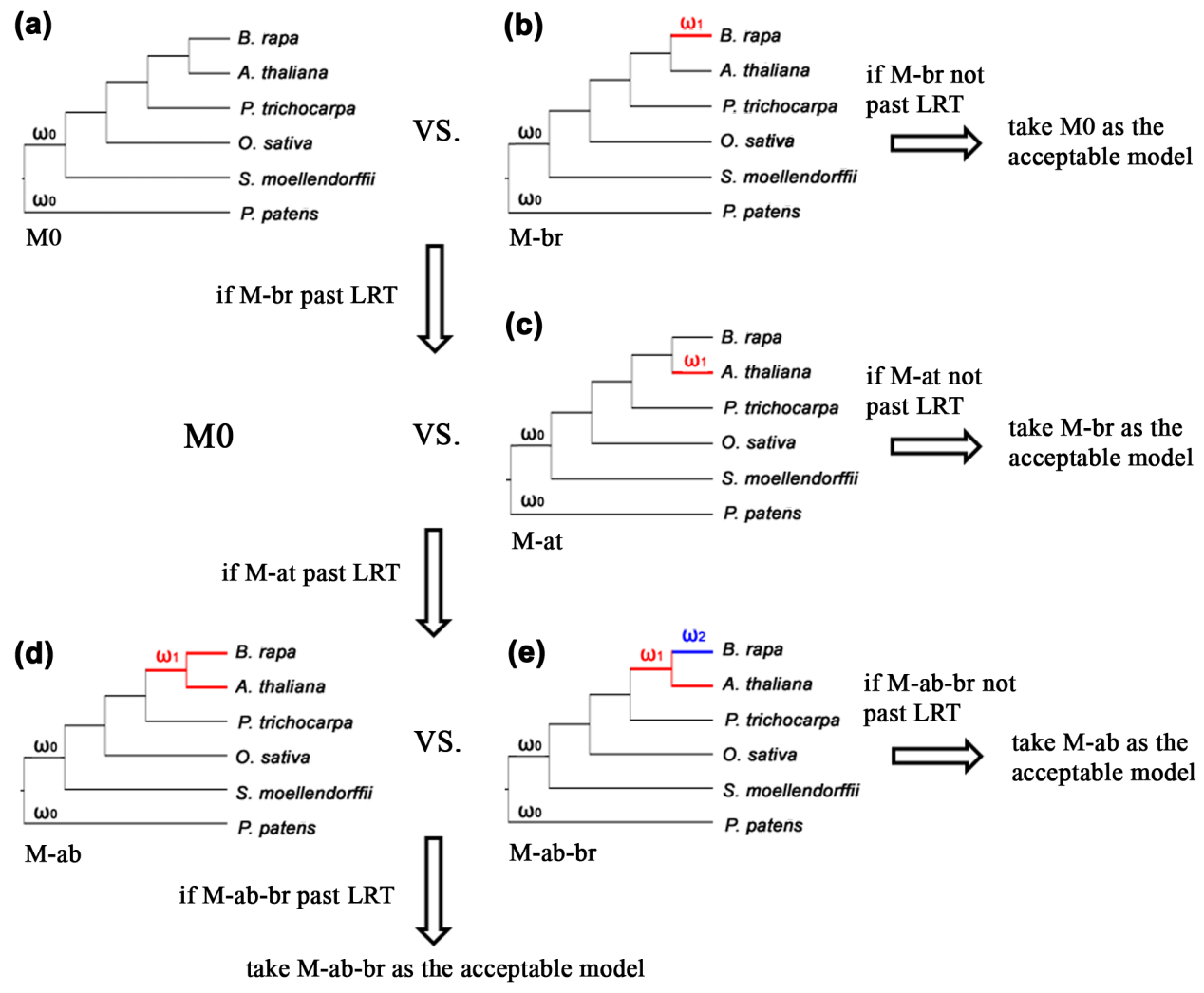
**
